# Redox Cascade in Chicken Skeletal Muscle: SELENOT Suppression in Selenium Deficiency Triggers Disulfidptosis via mtROS‐NADPH Dysregulation

**DOI:** 10.1002/advs.202507283

**Published:** 2025-09-15

**Authors:** Huanyi Liu, Hao Wu, Ziyu Zhang, Shiwen Xu, Cong Zhou, Tong Xu

**Affiliations:** ^1^ College of Veterinary Medicine Northeast Agricultural University Harbin 150030 P. R. China; ^2^ Key Laboratory of the Provincial Education Department of Heilongjiang for Common Animal Disease Prevention and Treatment College of Veterinary Medicine Northeast Agricultural University Harbin 150030 P. R. China; ^3^ Laboratory of Embryo Biotechnology College of Life Science Northeast Agricultural University Harbin 150030 P. R. China

**Keywords:** disulfidptosis, NADPH metabolism, oxidative stress, SELENOT, skeletal muscle atrophy

## Abstract

Skeletal muscle atrophy in poultry is characterized by reduced muscle mass and fiber quantity, leading to substantial economic losses in poultry production worldwide. Selenium is an essential trace element that maintains muscle integrity; however, the mechanisms linking Se deficiency to muscle injury remain unclear. Selenoprotein T (SELENOT) is a key regulator of cellular redox homeostasis that has not been fully characterized in skeletal muscles. Se deficiency downregulates SELENOT expression, increases oxidative stress, and induces skeletal muscle atrophy via disulfidase pathways. SELENOT deficiency impaired mitochondrial respiratory chain function, causing mitochondrial reactive oxygen species (mtROS) overproduction, glucose metabolism reprogramming, and Nicotinamide Adenine Dinucleotide Phosphate (NADPH) metabolism disruption. These changes result in cysteine accumulation and disulfidptosis, which lead to abnormal actin disulfide bonding. TEMPO‐mediated mtROS inhibition or NADPH supplementation partially rescues Se‐deficiency‐induced muscle atrophy. SELENOT overexpression alleviates the redox imbalance, NADPH dysfunction, disulfidptosis, and myotube atrophy in Se‐deficient cells, whereas rotenone‐induced mtROS activation or BAY‐876‐mediated NADPH inhibition reverses these protective effects. The SELENOT/mtROS/NADPH axis is crucial for Se‐deficiency‐induced muscle atrophy. This study provides mechanistic insights into muscle‐wasting disorders and potential therapeutic targets.

## Graphical abstract

Se deficiency downregulates SELENOT, disrupting mitochondrial function and elevating mtROS to induce glucose metabolic reprogramming, NADPH depletion, cysteine accumulation, and disulfidptosis‐mediated actin cytoskeleton collapse in chicken skeletal muscle. Interventions like SELENOT overexpression, mtROS inhibition, or NADPH supplementation partially restore redox homeostasis and NADPH function, inhibiting disulfidptosis to alleviate atrophy. These insights highlight the SELENOT/mtROS/NADPH axis for muscle wasting therapies.

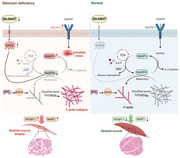

## 1 Introduction

Skeletal muscle is the largest tissue in adult animals, accounting for 40–50% of body weight.^[^ ^1^ ^]^ Skeletal muscle plays an important role in maintaining various basic physiological functions, such as postural support, strength generation, and exercise performance. Skeletal muscle atrophy is a serious health problem^[^ ^2^ ^]^ characterized by the progressive loss of muscle mass and strength, being common in various pathological processes, such as disuse, muscular dystrophy, and nerve compression injuries.^[^ ^3^ ^]^ Oxidative stress is a key factor leading to muscle atrophy and is involved in activating metabolic reprogramming and the ubiquitin‐related proteasome system in skeletal muscles.^[^ ^4^ ^]^ Lipopolysaccharides can disrupt mitochondrial function and promote the abnormal accumulation of mitochondrial reactive oxygen species (mtROS), leading to muscle atrophy.^[^ ^5^ ^]^ Metabolic reprogramming appears to be associated with mitochondrial dysfunction in skeletal muscle diseases.^[^ ^4^, ^6^ ^]^ Adaptive metabolic reprogramming caused by mitochondrial dysfunction occurs in patients with pulmonary arterial hypertension, accelerating muscle atrophy and reducing exercise endurance.^[^ ^7^ ^]^ Nicotinamide Adenine Dinucleotide Phosphate (NADPH) metabolism plays a crucial role in maintaining skeletal muscle mass and function.^[^ ^8^ ^]^ The NADPH levels in the skeletal muscle in quails fed niacin‐free feed were substantially lower than those in controls; the quails experienced muscle atrophy, inhibited growth, and reduced activity.^[^ ^9^ ^]^ No effective therapeutic intervention is currently available for treating muscle atrophy. Consequently, innovative strategies and therapeutic approaches must be developed to mitigate or prevent the progressive loss of skeletal muscle mass.

Disulfidiptosis is a form of regulated cell death marked by aberrant disulfide bond accumulation, which triggers cytoskeletal collapse^.[^ ^10^ ^]^ Disulfidptosis is caused by NADPH depletion, inducing disulfide stress that selectively disrupts cytoskeletal proteins.^[^ ^11^ ^]^ Disulfidptosis occurs via distinct redox mechanisms from those of ferroptosis, which involves Glutathione Peroxidase 4 (GPX4)‐mediated iron‐dependent lipid peroxidation.^[^ ^12^ ^]^ Although both are regulated by redox mechanisms, ferroptosis drives death through lipid peroxide buildup and membrane rupture, whereas disulfidptosis drives death via protein–disulfide bonding and cytoskeletal failure.

Cysteine is an important precursor for synthesizing GSH in cells. Cysteine transport is dependent on the Solute Carrier Family 7 Member 11 (SLC7A11) transporter, which indirectly participates in synthesizing antioxidant enzymes and inhibits oxidative stress.^[^ ^13^ ^]^ Cellular cysteine must be reduced to cysteine, which requires large amounts of NADPH, to avoid toxic cysteine accumulation.^[^ ^14^ ^]^ Metabolic reprogramming usually occurs when cells experience high levels of oxidative stress, resulting in an insufficient glucose supply and NADPH depletion, leading to abnormally high accumulation of intracellular disulfide.^[^ ^14^, ^15^ ^]^ This disulfide not only reduces the antioxidant capacity of cells but also induces abnormal cross‐linking of the actin cytoskeleton, destroying its structure and function.^[^ ^10^, ^16^ ^]^ An increase in reactive oxygen species (ROS) levels in the basal cells of mice promotes pentose phosphate pathway (PPP) metabolic disorder and high expression levels of SLC7A11, leading to disulfidptosis, and ultimately skin psoriasis.^[^ ^17^ ^]^ Fang et al. found that ferritin H deficiency promoted cardiomyopathy through SLC7A11‐mediated disulfidptosis, supporting a role of disulfidptosis in cardiomyopathy development.^[^ ^18^ ^]^ Exposure to carbon quantum dots induces metabolic reprogramming and NADPH depletion by elevating ROS levels, leading to disulfidptosis and neuronal disorders.^[^ ^19^ ^]^ The role of disulfide ptosis in many diseases has been partially characterized; however, the effects of disulfidptosis, on muscle atrophy are unknown.

Se is an essential trace element found in vertebrates. Se is involved in a variety of biological processes, such as antioxidant activity, enhancing immunity, and regulating tissue metabolism, as an important part of selenoprotein biosynthesis.^[^ ^20^ ^]^ The Se concentration in muscle is positively correlated with muscle mass and strength. Se deficiency is frequently associated with clinical manifestations such as muscle pain, proximal weakness, and chronic fatigue.^[^ ^21^ ^]^ Se levels affect the fate of skeletal muscle cells, and Se deficiency induces skeletal muscle atrophy through oxidative stress.^[^ ^1^, ^22^ ^]^

Selenoprotein T (SELENOT) is a 22 kDa membrane‐associated selenoprotein that inhibits oxidative stress and maintains intracellular homeostasis.^[^ ^23^ ^]^ Most studies on SELENOT have focused on its role in cardiovascular disease, confirming its influence on cell structure and adhesion properties.^[^ ^24^ ^]^ The muscle‐related functions of selenoproteins (SELENOW, GPx3, and GPx4) are well‐understood; however, the function of SELENOT in skeletal muscles, especially in muscle atrophy, has been less studied.

The chicken is an ideal animal model for studying skeletal muscle, as the developmental anatomy of chickens is similar to that of mammals such as humans.^[^ ^25^ ^]^ We established a Se‐deficient chicken model to study the impact of Se deficiency on selenoprotein levels in skeletal muscle and the role of SELENOT. We confirmed the role of NADPH metabolism and cytoskeletal proteins in the skeletal muscle atrophy caused by SELENOT deficiency through comprehensive transcriptome and proteomics analyses. The in vitro results showed that SELENOT deficiency induced a large amount of mtROS to interfere with NADPH synthesis, induced the disulfidptosis of skeletal muscle cells, and finally caused skeletal muscle atrophy with the collapse of the actin cytoskeleton. We determined the mechanism through which Se‐deficient skeletal muscle atrophy in chickens is dependent on disulfidptosis, finding that the SELENOT/mtROS/NADPH pathway could be targeted for treating skeletal muscle atrophy.

## 2 Results

### 2.1 SELENOT Expression Levels

**Figure 1:**
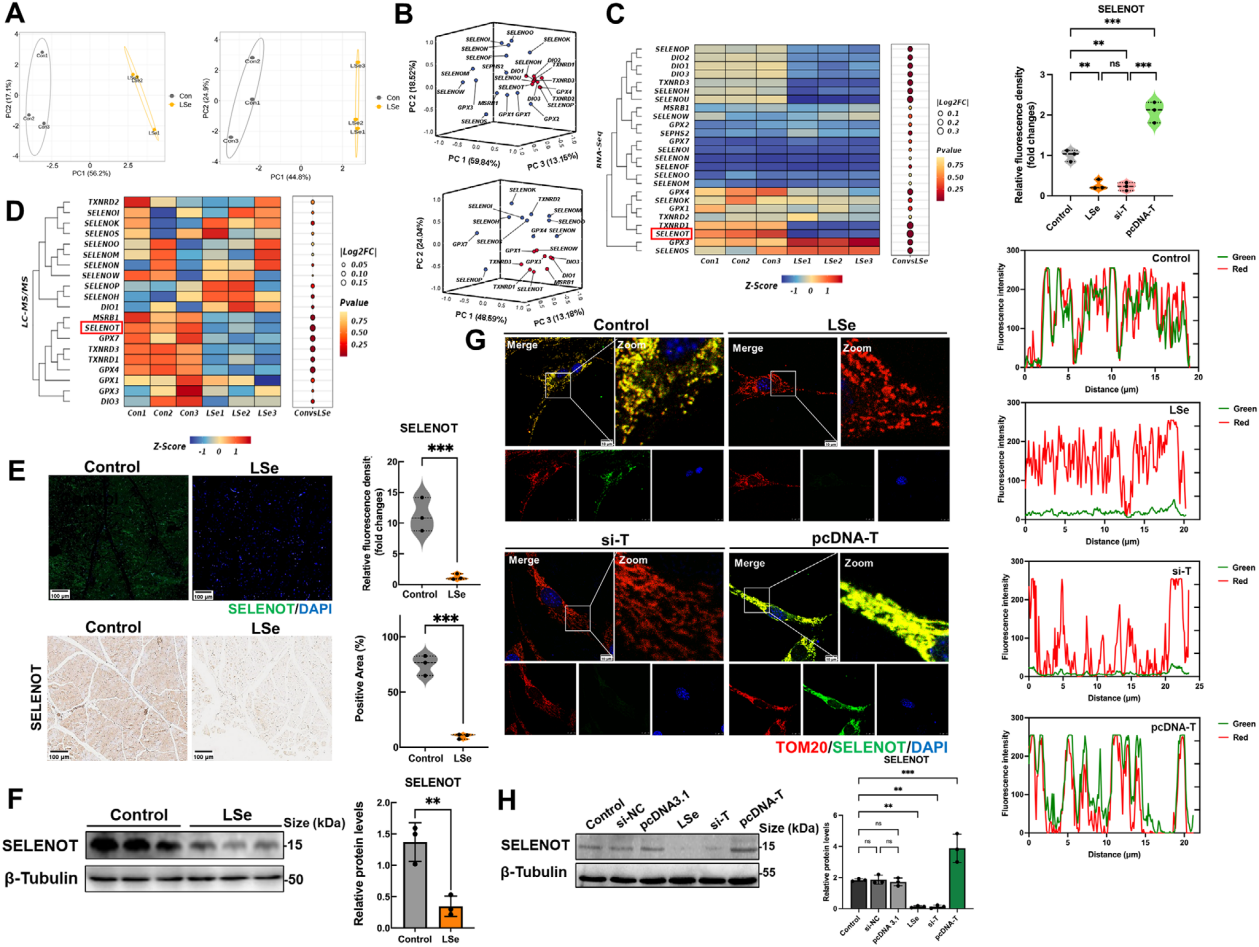
SELENOT is downregulated in the skeletal muscle of Se deficient chickens. A) Correlation analysis of overall Se transcriptome and proteome between Control group and Low Se (LSet) group. B) Se transcriptome and proteome principal component analysis. C) The expression of selenoprotein mRNA in the sequencing results. The size of the circle represents the Log2FC value of the gene. The depth of the color represents the *p*‐value of the gene. Z‐scores of data sets were standardized to −2–2. D) The expression of Se containing proteins in the sequencing results. The size of the circle represents the Log2FC value of the protein. The depth of the color represents the *p*‐value of the protein. Z‐scores of data sets were standardized to −2–2. E) Skeletal muscle selenoprotein T (SELENOT) immunofluorescence and immunohistochemistry staining. Scale bar: 100 µm. *p*‐values were measured by *t*‐test. *n* = 3. F) Western Blot analysis of SELENOT in skeletal muscle. *p*‐values were measured by *t*‐test. *n* = 3. G) SELENOT and TOM20 co localization staining of skeletal muscle cells in vitro. Scale bar: 10 µm. *p*‐values were measured by One‐Way ANOVA. *n* = 3. H) Western Blot analysis of SELENOT in vitro skeletal muscle cells. *p*‐values were measured by One‐Way ANOVA. *n* = 3. The data are shown as the mean ± SDs. ^*^ *p* < 0.05, ^**^ *p* < 0.01, and ^***^ *p* < 0.001.

The biological functions of Se are primarily mediated by selenoproteins. We investigated the impact of Se deficiency on the selenoprotein expression levels in skeletal muscles using conducted transcriptomic and proteomic analyses. The results of correlation analyses revealed considerable differences in the selenoprotein expression profiles between chickens fed normal and Se‐deficient diets (**Figure** **1**A). We identified the key selenoproteins affected by Se deficiency using principal component analysis. The results revealed distinct clustering patterns of 10 selenoprotein transcripts and 9 selenoproteins in 3D space (Figure 1B). Se deficiency resulted in the downregulation of the levels of 11 selenoprotein transcripts and 8 selenoproteins in the skeletal muscle (Figure 1C,D). The transcriptional and protein levels of SELENOT were the most strongly downregulated among these selenoproteins. SELENOT expression levels were markedly downregulated in the skeletal muscles of Se‐deficient chickens compared with those in the controls, indicating the physiological relevance of SELENOT. We further confirmed the differences in SELENOT expression levels in the skeletal muscle between the groups of chickens using immunofluorescence (IF), immunohistochemistry (IHC), and Western blot analyses. A Se‐deficient diet reduced the SELENOT expression levels in the skeletal muscle of chickens (Figure 1E,F). We analyzed the effect of Se deficiency on skeletal muscle in vitro by extracting primary chicken embryo myoblasts. Myoblast differentiation was induced using 2% horse serum for seven days to form myotubes (Figure S1, Supporting Information). SELENOT is a selenoprotein in the mitochondria, and we used laser confocal microscopy to find that SELENOT and the mitochondrial outer membrane protein TOM20 colocalized in muscle cells (Figure 1G). In addition, SELENOT protein expression was knocked down and overexpressed using si‐T and pcDNA‐T plasmids in muscle cells, respectively. The SELENOT fluorescence intensity and protein expression in the mitochondria were considerably lower after SELENOT knockdown, consistent with the changes in SELENOT levels after the chickens were fed a Se‐deficient diet. The SELENOT fluorescence intensity and protein expression in the mitochondria were markedly higher after SELENOT overexpression (Figure 1H).

### 2.2 Actin Cytoskeleton and Metabolic Processes Influenced by SELENOT Downregulation under Se Deficiency

**Figure 2:**
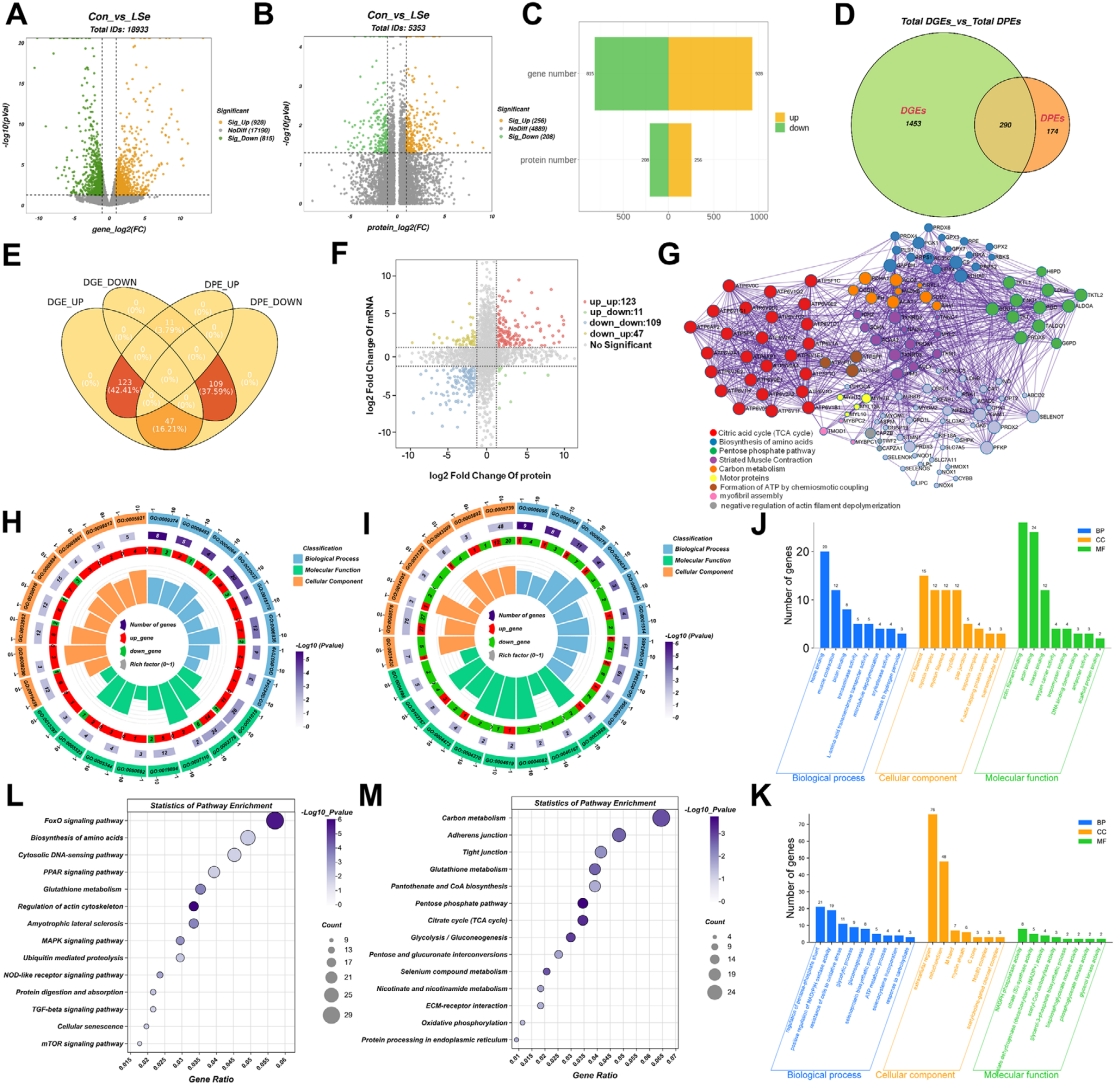
SELENOT exerts its effects by affecting the actin cytoskeleton and metabolic processes. A) Volcanic maps of Differentially expressed genes (DEGs) in skeletal muscle of chickens in the Control group and LSe group. B) Volcanic maps of Differentially expressed proteins (DEPs) in skeletal muscle of chickens in the Control group and LSe group. C) Comparative analysis of DEGs and DEPs. D,E) Venn plot shows the co‐expression of DEGs and DEPs. F) Nine‐quadrant diagram of DEGs and DEPs G) Co‐expression network of DEGs and DEPs H) GO enrichment circle plot of co‐upregulation of DEGs and DEPs I) GO enrichment circle plot of co‐downregulation of DEGs and DEPs J) GO analysis shows enrichment of co‐upregulation of DEGs and DEPs functional categories in skeletal muscle. K) GO analysis shows enrichment of co‐downregulation functional categories in skeletal muscle. L) KEGG pathway enrichment analysis of co‐upregulated DEGs and DEPs in Skeletal Muscle M) KEGG pathway enrichment analysis of co‐downregulated DEGs and DEPs in Skeletal Muscle.

We investigated the response of the skeletal muscle of Se‐deficient chickens to SELENOT downregulation by analyzing the RNA and protein sequencing of the skeletal muscle tissue. We detected 18933 genes, including 1743 differentially expressed genes (DEGs), of which 928 and 815 were up‐ and downregulated, respectively (**Figure** **2**A). The DEGs were identified at thresholds of *P*<0.05 and | Log2FC| > 1. A total of 464 differentially expressed proteins (DEPs) were identified, of which 256 and 208 were significantly up‐ and downregulated in the skeletal muscle of chickens fed a Se‐deficient diet, respectively (Figure 2B). DEPs were identified at *P*<0.05, | Log2FC| > 1. Among these, 290 DEPs and DEGs were significantly regulated at both the gene and protein levels (Figure 2C). The results of Venn analysis showed that the expression trends of 232 DEPs (123 and 109 up‐ and downregulated, respectively) were consistent between gene and protein levels (Figure 2D,E). The nine‐quadrant diagram in Figure 2F illustrates the correlation between the transcriptomic and proteomic data, revealing that 123 and 109 mRNAs and their corresponding proteins were consistently up‐ and downregulated, respectively; 11 mRNAs were upregulated while their proteins were downregulated; and 47 mRNAs were downregulated while their proteins were upregulated. We then constructed a coexpression network for the mRNAs and proteins with consistent expression trends (Figure 2G) using weighted gene co‐expression network analysis, which highlighted the associations of coexpressed genes and proteins with the tricarboxylic acid (TCA) cycle, PPP, and cytoskeleton protein pathway. Gene Ontology (GO) functional analysis and Kyoto Encyclopedia of Genes and Genomes (KEGG) pathway analysis were conducted to obtain further information on the functions of the DEGs and DEPs. The GO enrichment analysis of the co‐upregulated genes and proteins revealed that the majority of DEGs and DEPs were significantly enriched in pathways associated with the actin cytoskeleton, muscle atrophy, and oxidative stress (Figure 2H,J). We found that the following terms were enriched: microtubule depolymerization (GO: 0007019); muscle contraction (GO: 0006936); myosin complex (GO: 0016459); F‐actin capping protein complex (GO: 0008290); scaffold protein binding (GO: 0097110); actin binding (GO: 0003779); response to hydrogen peroxide (GO: 0042542). Furthermore, the co‐downregulated DEGs and DEPs were primarily enriched in the synthesis and metabolism of carbon‐containing compounds, mainly involving alterations in sugar and NADPH metabolism, such as regulation of pentose phosphate shun (GO: 0043456); positive regulation of NAD(P)H oxidase activity (GO: 0033864); glycerol‐3‐phosphate biosynthetic process (GO: 0046167); response to carbohydrate (GO: 0009743); glycolytic process (GO: 0006096); NADPH phosphatase activity (GO: 0102757). We also found that the resistance of cells to oxidative stress (GO: 0006979) and their ability to synthesize Se‐containing proteins (GO: 0097056) were enriched in the co‐downregulated DEGs and DEPs. The KEGG results showed that the co‐upregulated DEGs and DEPs were significantly enriched in pathways associated with the oxidative stress response, cytoskeleton disruption, and muscle pathology, including regulating the actin cytoskeleton (ko04810), FoxO signaling pathway (ko04068), amyotrophic lateral sclerosis (ko05014), glutathione metabolism (ko00480), and MAPK signaling pathway (ko04010) (Figure 2L). In contrast, the co‐downregulated DEGs and DEPs were primarily enriched in metabolic pathways critical for producing energy and synthesizing NADPH, such as the pentose phosphate pathway (ko00030), citrate cycle (TCA cycle) (ko00020), glycolysis/gluconeogenesis (ko00010), carbon metabolism (ko01200), and Se compound metabolism (ko00450) (Figure 2M). These results demonstrate that SELENOT downregulation during Se deficiency disrupts the actin cytoskeleton organization and metabolic homeostasis in skeletal muscle cells.

### 2.3 SELENOT Maintained Mitochondrial Redox Homeostasis in Chicken Skeletal Muscle

**Figure 3:**
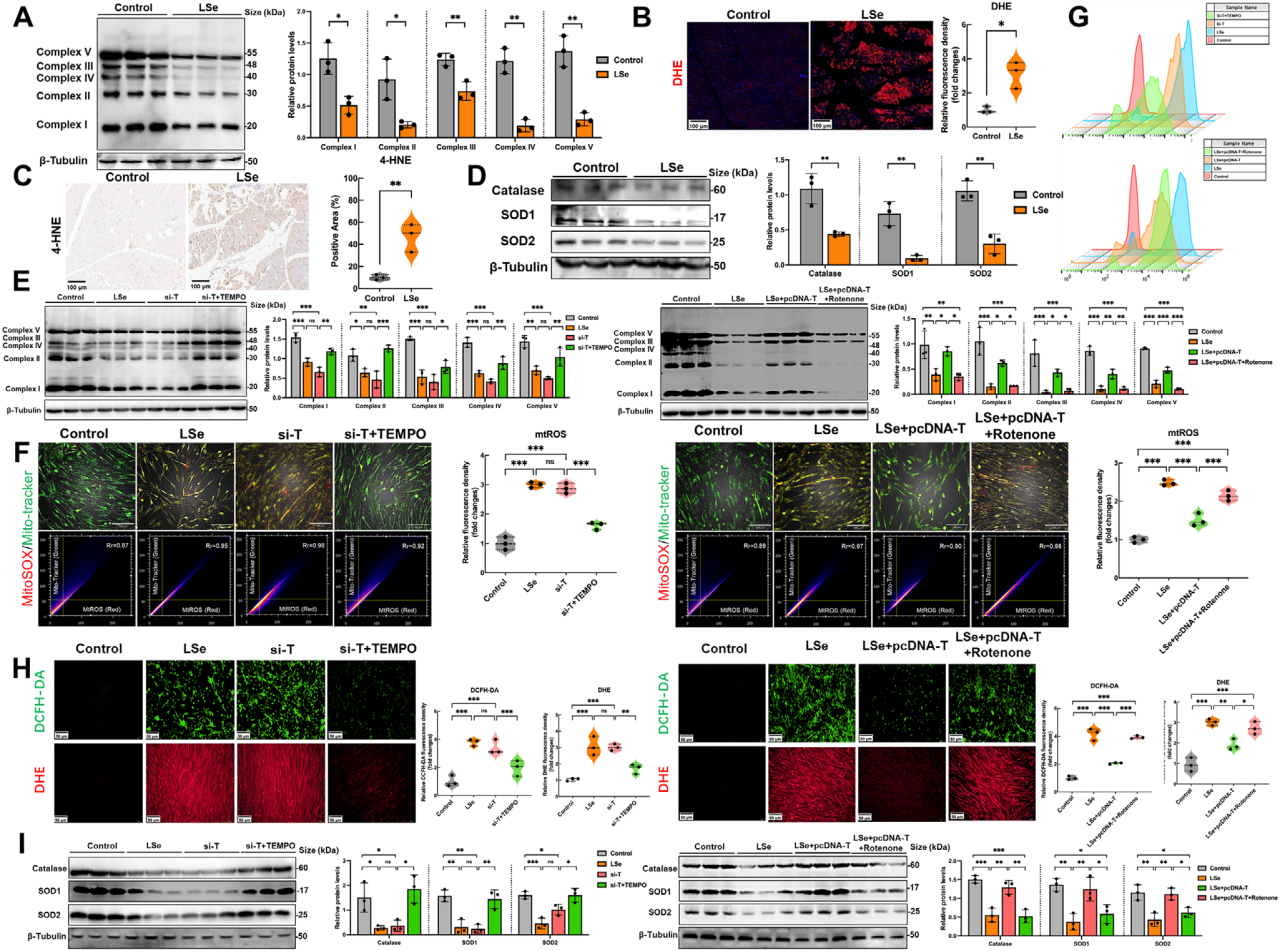
SELENOT maintains skeletal muscle redox homeostasis through mitochondrial respiratory chain complex (MRCC). A) Western blotting analysis of MRCC in skeletal muscle. *p*‐values were measured by t‐test. *n* = 3. B) Skeletal muscle Dihydroethidium (DHE) fluorescence staining. Scale bar: 100 µm. *p*‐values were measured by *t*‐test. *n* = 3. C) Skeletal muscle 4‐Hydroxynonenal (4‐HNE) immunohistochemical staining. Scale bar: 100 µm. *p*‐values were measured by *t*‐test. *n* = 3. D) Western Blot analysis of antioxidant enzymes in skeletal muscle. *p*‐values were measured by *t*‐test. *n* = 3. E) Western Blot analysis of MRCC in skeletal muscle cells in vitro. *p*‐values were measured by One‐Way ANOVA. *n* = 3. F) Mitochondrial reactive oxygen species (mtROS) fluorescence staining of skeletal muscle cells in vitro. Scale bar: 100 µm. *p*‐values were measured by One‐Way ANOVA. *n* = 3. G) mtROS analysis results flow cytometry. *n* = 3. H) ROS and DHE fluorescence staining of skeletal muscle cells in vitro. Scale bar: 50 µm. *p*‐values were measured by One‐Way ANOVA. *n* = 3. I) Western Blot analysis of antioxidant enzymes in skeletal muscle cells in vitro. *p*‐values were measured by One‐Way ANOVA. *n* = 3. The data are shown as the mean ± SDs. ^*^ *p* < 0.05, ^**^ *p* < 0.01, and ^***^ *p* < 0.001.

We determined the precise role of SELENOT in the mitochondria by assessing the alterations in the expression and activity of mitochondrial respiratory chain complexes in the skeletal muscle of chickens. The results of Western blotting and enzymatic activity assays showed that the expression and function of mitochondrial respiratory chain complexes were impaired in the skeletal muscles of SELENOT‐deficient chicken (**Figure** **3**A; Figure S2, Supporting Information). The appearance of a red signal upon dihydroethidium (DHE) staining indicated ROS accumulation, which was associated with an increase in oxidative stress levels. An important lipid peroxidation marker is 4‐HNE. The DHE and 4‐HNE staining of chicken skeletal muscles showed that ROS levels were considerably higher in SELENOT‐deficient muscles than in the control group (Figure 3B,C). The levels of the antioxidant enzymes (CAT, SOD1, and SOD2) in the skeletal muscle of SELENOT‐deficient chickens were substantially lower than those in the control group (Figure 3D), indicating the SELENOT‐deficient chickens were less resistant to oxidative stress.

We investigated the role of SELENOT in regulating oxidative stress in muscle cells in vitro. SELENOT knockdown disrupted the expression of mitochondrial respiratory chain complexes and reduced their enzymatic activities, mirroring the effects of Se deficiency. This disruption was partially rescued through administering the mtROS inhibitor TEMPO. Conversely, SELENOT overexpression alleviated both the Se‐deficiency‐induced suppression of mitochondrial respiratory chain complex expression and its functional activities, which were abolished by the mtROS activator rotenone (Figure 3E; Figure S3, Supporting Information). The flow cytometry and MitoSOX staining results revealed that SELENOT knockout induced mitochondrial ROS accumulation, which was attenuated by TEMPO treatment. Furthermore, SELENOT overexpression reduced the Se‐deficiency‐induced mitochondrial increase in ROS levels, whereas rotenone administration abolished this protective effect (Figure 3F,G). The changes in the total intracellular ROS levels were consistent with the changes in the mtROS levels (Figure 3H).

We examined the effects of SELENOT on intracellular antioxidant enzymes in muscle cells, as the levels of antioxidant enzymes were downregulated in the skeletal muscles of chickens lacking SELENOT. The antioxidant enzyme levels were lower in SELENOT‐knockout and Se‐deficient muscle cells than in the control cells, which were partially restored by adding TEMPO. SELENOT overexpression increased the low levels of antioxidant enzymes caused by Se deficiency, whereas rotenone reversed the levels (Figure 3I).

In summary, SELENOT is closely associated with the mitochondrial redox homeostasis in the skeletal muscle of chickens. SELENOT deficiency exacerbates the disruption of the mitochondrial respiratory chain and an increase in mtROS levels, whereas SELENOT overexpression helps maintain normal mitochondrial respiratory chain complex function and inhibits oxidative stress.

### 2.4 SELENOT Regulated NADPH Metabolism in Chicken Skeletal Muscle Through mtROS

**Figure 4:**
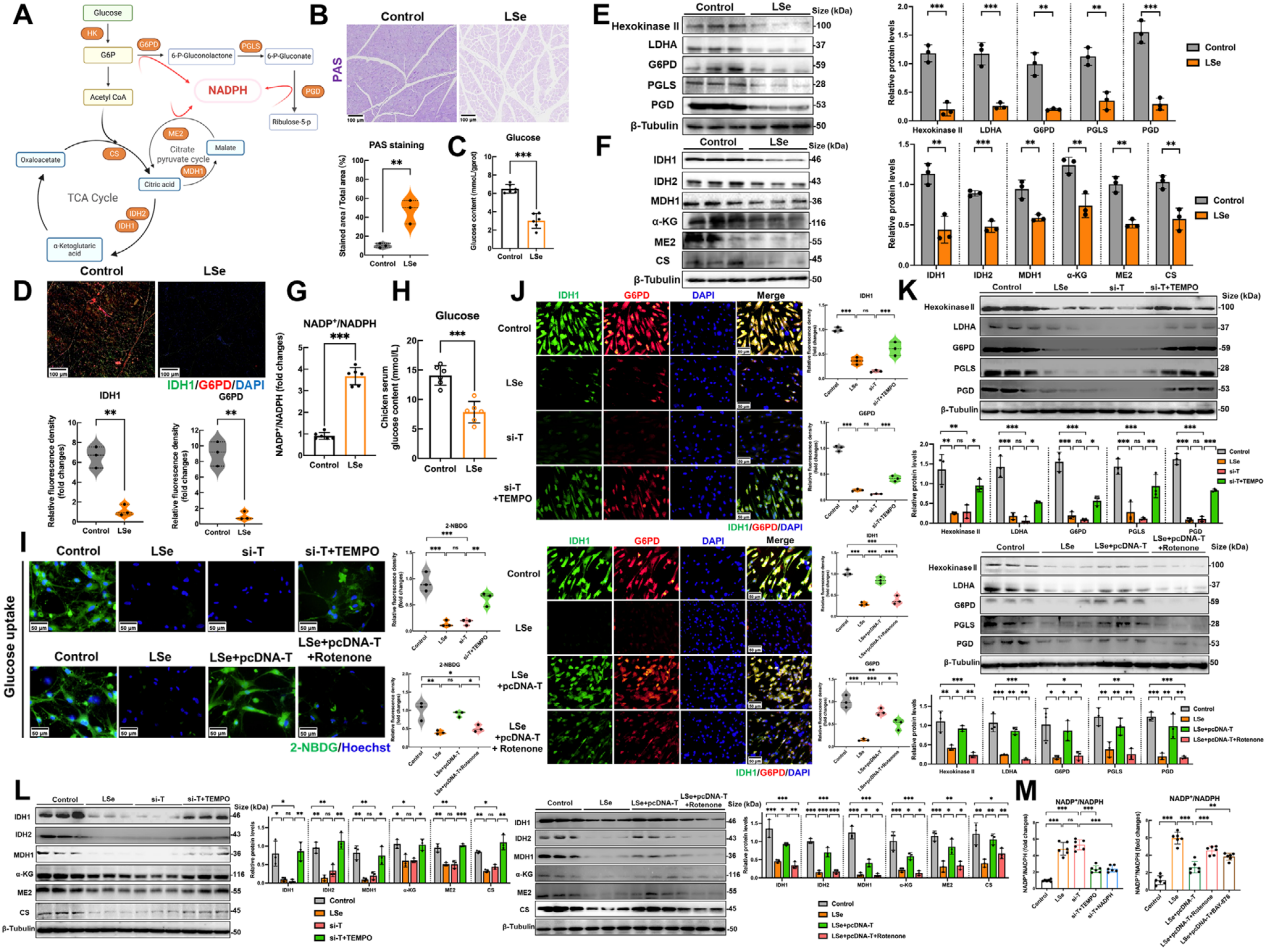
SELENOT/mtROS regulates NADPH synthesis and metabolism in skeletal muscle cells. A) Schematic diagram of intracellular NADPH synthesis pathway. Image crated with BioRender.com, with permission. B) Skeletal muscle PAS staining. Scale bar: 100 µm. *p*‐values were measured by *t*‐test. *n* = 3. C) Skeletal muscle glucose content. *p*‐values were measured by *t*‐test. *n* = 6. D) Skeletal muscle IDH1 and G6PD fluorescence staining. Scale bar: 100 µm. *p*‐values were measured by *t*‐test. *n* = 3. E) Western Blot analysis of key enzymes involved in the PPP pathway in skeletal muscle. *p*‐values were measured by *t*‐test. *n* = 3. F) Western Blot analysis of key enzymes involved in the TCA cycle in skeletal muscle. *p*‐values were measured by *t*‐test. *n* = 3. G) The changes of NADP^+^/NADPH in skeletal muscle. *p*‐values were measured by t‐test. n = 6. H) Chicken serum glucose content. *p*‐values were measured by *t*‐test. *n* = 6 (I) 2‐NBDG staining of skeletal muscle cells in vitro. Scale bar: 50 µm. *p*‐values were measured by One‐Way ANOVA. *n* = 3. J) Fluorescence staining of IDH1 and G6PD in skeletal muscle cells in vitro. Scale bar: 50 µm. *p*‐values were measured by One‐Way ANOVA. *n* = 3. K) Western Blot analysis of key enzymes involved in the PPP pathway in skeletal muscle cells. *p*‐values were measured by One‐Way ANOVA. *n* = 3. L) Western Blot analysis of key enzymes involved in the TCA cycle in skeletal muscle cells. *p*‐values were measured by One‐Way ANOVA. *n* = 3. M) The changes of NADP^+^/NADPH in skeletal muscle cells. *p*‐values were measured by One‐Way ANOVA. *n* = 6. Error bars indicate the SEM. The data are shown as the mean ± SDs. ^*^ *p* < 0.05, ^**^ *p* < 0.01, and ^***^ *p* < 0.001.

Glucose is an important substrate for cellular energy metabolism. NADPH is continuously produced through the PPP, tricarboxylic acid, and citrate pyruvate pathways (**Figure** **4**A). We performed PAS and IDH1/G6PD staining of chicken skeletal muscle to characterize the role of SELENOT in skeletal muscle during NADPH metabolism. SELENOT deficiency reduced the synthesis of glucose and glycogen in chicken skeletal muscle compared with those in the control group. IDH1 and G6PD are key enzymes of the TCA cycle and pentose bypass pathway; their syntheses were also inhibited by SELENOT deficiency (Figure 4B–D). Hexokinase II, LDHA, G6PD, PGLS, and PGD are enzymes involved in the PPP; their levels were downregulated in the skeletal muscle of SELENOT‐deficient chickens (Figure 4E). IDH1, IDH2, MDH1, α‐KG, ME2, and CS are enzymes involved in the TCA cycle and the citrate pyruvate pathway; their levels were similarly reduced by the lack of SELENOT (Figure 4F). The NADPH levels in SELENOT‐deficient skeletal muscle in chickens were than those in the control group (Figure 4G). This indicated that SELENOT deficiency inhibited NADPH anabolism in the skeletal muscle of chickens.

We measured the serum glucose levels in chicken serum, which were under Se‐deficient conditions than in the control group (Figure 4H). We studied the intrinsic mechanism affecting NADPH synthesis in vitro in depth as SELENOT regulates mtROS production in muscle cells. Specifically, our temporal analysis revealed that mtROS levels increased within 3 h of SELENOT knockdown, whereas NADPH depletion was evident only after 12–24 h. Early‐phase (3 h) treatment with TEMPO suppressed mtROS accumulation and prevented subsequent NADPH depletion, whereas late‐phase (12 h) NADPH supplementation failed to mitigate mtROS overproduction, establishing elevated mtROS levels as the initiating event (Figure S4A–C, Supporting Information). We found that Se deficiency or SELENOT knockout reduced the glucose content and IDH1/G6PD expression in muscle cells compared with those in the control, and inhibiting intracellular mtROS production using TEMPO effectively alleviated these changes. In addition, SELENOT overexpression inhibited the decrease in glucose content and IDH1/G6PD expression caused by Se deficiency; these changes were reversed by the addition of the mtROS activator, rotenone (Figure 4I,J).

We investigated the effect of SELENOT on glucose metabolism in the skeletal muscle cells of chickens by assessing both the oxygen consumption rate (OCR, reflecting oxidative phosphorylation/OXPHOS) and extracellular acidification rate (ECAR, indicating glycolytic flux). Our data revealed that either Se deficiency or SELENOT knockdown impaired mitochondrial OXPHOS capacity while suppressing glycolytic activity, which was evidenced by the reductions in basal glycolysis, glycolytic capacity, and the glycolytic reserve (Figure S5, Supporting Information). These metabolic perturbations were substantially reversed by TEMPO treatment. SELENOT overexpression effectively ameliorated the Se‐deficiency‐induced impairment of OXPHOS and glycolysis. Cotreatment with the mtROS activator, rotenone, exacerbated these metabolic dysfunctions. The expression levels of key enzymes involved in NADPH generation and the intracellular NADPH content were also inhibited in Se‐deficient or SELENOT‐knockdown cells. The PPP, hexokinase II, LDHA, G6PD, PGLS, PGD, TCA cycle, and citrate pyruvate pathway, IDH1, IDH2, MDH1, α‐KG, ME2, and CS were also inhibited. These changes were reversed by adding TEMPO. SELENOT overexpression effectively alleviated the inhibition of key enzymes in the PPP, TCA cycle, and citrate pyruvate pathway in cells under Se deficiency and restored intracellular NADPH levels. Adding rotenone produced opposite results to SELENOT overexpression (Figure 4K–M). Overall, SELENOT deficiency inhibited NADPH anabolism in the skeletal muscle of chickens through mtROS, effectively reducing mtROS levels in the skeletal muscle, which was conducive to NADPH synthesis.

### 2.5 SELENOT Alleviated SLC7A11^high^ and Disulfidptosis Caused by NADPH Deficiency

**Figure 5:**
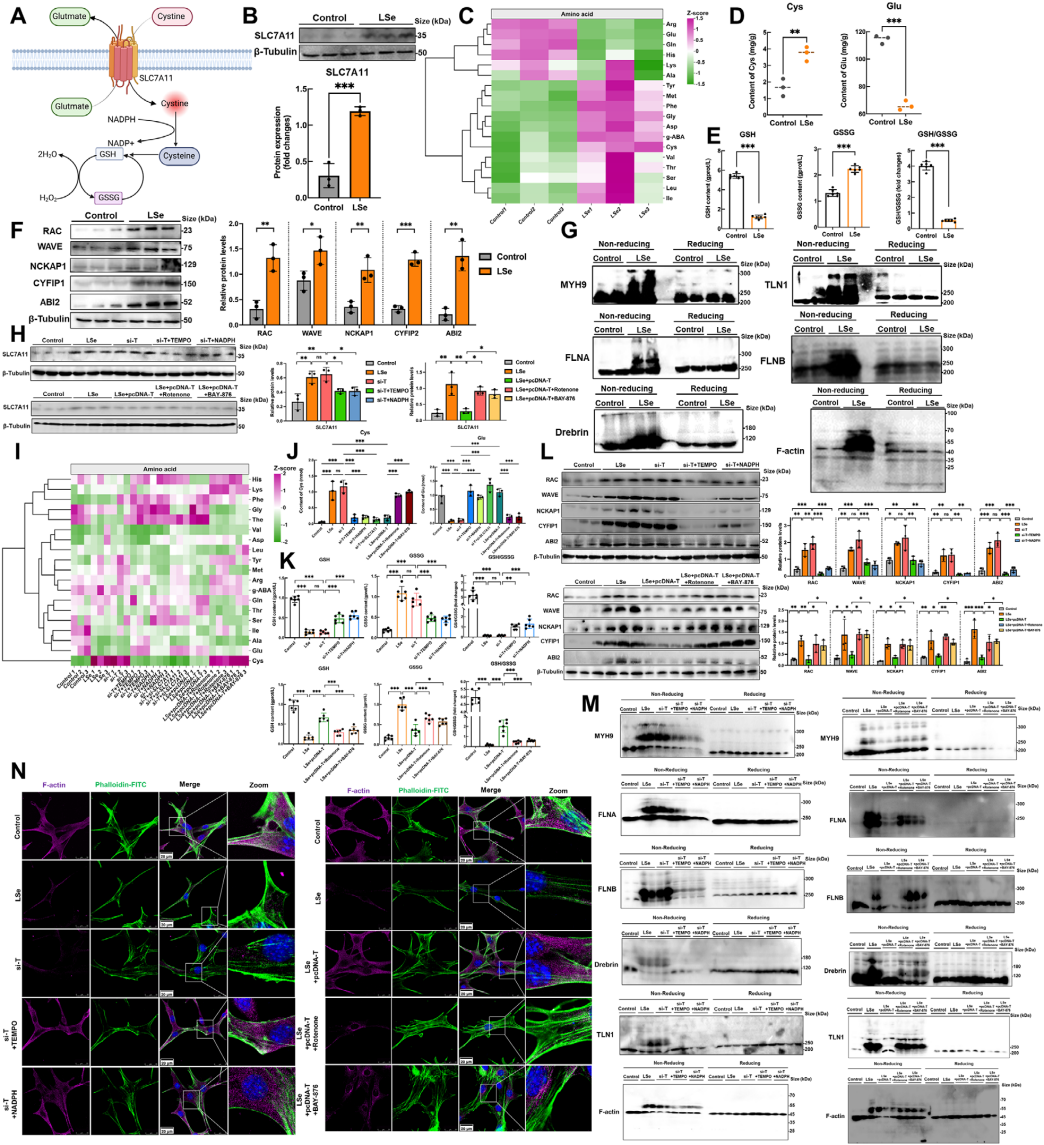
SELENOT/mtROS/NADPH axis mediates disulfidptosis in chicken skeletal muscle. A) Schematic diagram of SLC7A11 transporter protein operation. Image created with BioRender.com, with permission. B) Western Blot analysis of SLC7A11 in skeletal muscle. *p*‐values were measured by a *t*‐test. *n* = 3. C) Detection of amino acid content in skeletal muscle. Z‐scores of data sets were standardized to −1.5–1.5. D) The content of cystine and glutamate in skeletal muscle. *p*‐values were measured by a *t*‐test. *n* = 3. E) The content of GSH/GSSG in skeletal muscle. *p*‐values were measured by a *t*‐test. *n* = 6. F) Western Blot analysis of RAC/WAVE pathway‐related indicators in skeletal muscle. *p*‐values were measured by a *t*‐test. *n* = 3. G) Formation of disulfide bonds in actin cytoskeleton, *n* = 4. H) Western Blot analysis of SLC7A11 in skeletal muscle cells in vitro. *p*‐values were measured by One‐Way ANOVA. *n* = 3. I) Detection of amino acid content in skeletal muscle cells in vitro. Z‐scores of data sets were standardized to −2–2. J) The content of cystine and glutamate in skeletal muscle cells in vitro. *p*‐values were measured by One‐Way ANOVA. *n* = 3. (K) The content of GSH/GSSG in skeletal muscle cells in vitro. *p*‐values were measured by One‐Way ANOVA. *n* = 6. (L) Western Blot analysis of RAC/WAVE pathway‐related indicators in vitro skeletal muscle cells. *p*‐values were measured by One‐Way ANOVA. *n* = 3. M) Formation of disulfide bonds in the cytoskeleton of skeletal muscle cells, *n* = 4. N) F‐actin staining of skeletal muscle cells in vitro. Scale bar: 20 µm. The data are shown as the mean ± SDs. ^*^ *p* < 0.05, ^**^ *p* < 0.01, and ^***^ *p* < 0.001.

SLC7A11 participates in the extracellular uptake of cystine and the release of glutamate. SLC7A11 promotes glutathione synthesis by consuming NADPH and maintaining the cellular redox balance (**Figure** **5**A). We found that SELENOT deficiency increased the SLC7A11 protein level in the chicken skeletal muscle compared with that in the control (Figure 5B). Amino acid analysis showed that SELENOT‐deficient chickens accumulated substantially more cysteine and had considerably less glutamate in their muscle tissue than control chickens (Figure 5C,D). The ability of skeletal muscle to synthesize GSH was affected by SELENOT deficiency: GSH and GSH/GSSG levels were downregulated, and GSSG levels were higher (Figure 5E).

Cystine is a highly insoluble amino acid that triggers disulfidptosis when accumulated in large quantities. We examined the protein levels of the RAC/WAVE complexes associated with disulfidptosis. RAC/WAVE complexes formed in the SELENOT‐deficient chicken skeletal muscle, which was confirmed by the upregulated expression levels of RAC, WAVE, NCKAP1, CYFIP1, and ABI2 (Figure 5F). We then attempted to validate the formation of disulfide bonds in the actin cytoskeleton proteins induced by SELENOT deficiency using nonreducing Western blotting. The formation of disulfide bonds affects the electrophoretic mobility of proteins under nonreducing conditions. SELENOT deficiency resulted in the slower migration rates of various actin cytoskeleton proteins in diffuse bands under non‐reducing conditions. SELENOT deficiency did not induce the same migration pattern in these proteins under reducing conditions, indicating that these actin cytoskeletal proteins formed multiple intermolecular disulfide bonds under SELENOT‐deficient conditions (Figure 5G).

We conducted in‐depth studies in vitro to determine whether mtROS/NADPH were the direct factors through which SELENOT‐mediated disulfidptosis occurs in skeletal muscle cells. We found that SELENOT knockdown induced high levels of SLC7A11 in the skeletal muscle of the chickens compared with those in the control group, with a large amount of cystine being uptaken up by the cells. SELENOT knockdown also induced glutamate deficiency, inhibiting the GSH synthesis in the muscle cells, which is consistent with the state of muscle cells under Se deficiency. Adding TEMPO to inhibit mtROS, supplementing NADPH, or SLC7A11 knockdown helped reverse the above impacts in muscle cells, alleviating cystine accumulation and GSH/GSSG reduction (Figure S6A,B, Supporting Information). In addition, SELENOT overexpression effectively alleviated the high SLC7A11 levels as well as cystine accumulation and decreased the GSH/GSSG expression levels in muscle cells caused by Se deficiency. The mitigating effects of SELENOT overexpression were lost by adding the mtROS activator rotenone or NADPH inhibitor BAY‐876 (Figure 5H–K). We found that Se deficiency or SELENOT knockdown promoted the formation of disulfide bonds between the RAC/WAVE complex and its mediated actin cytoskeleton in muscle cells, similar to the in vivo results. Inhibiting mtROS, supplementing with NADPH, or SLC7A11 knockdown effectively prevented these changes, inhibiting disulfide bond formation (Figure 5L,M; Figure S6C,D, Supporting Information). SELENOT overexpression alleviated the formation of disulfide bonds between the RAC/WAVE complexes and their mediated actin cytoskeleton compared with that in the LSe group. Adding rotenone or BAY‐876 reversed this effect.

We also studied the state of the skeletal proteins after disulfide bonds formed to detect whether the muscle cells underwent disulfidptosis. The costaining of F‐actin with the membrane dye phalloidin showed that Se deficiency or SELENOT knockdown induced F‐actin detachment from the plasma membrane of the muscle cells. A normal cellular state was recovered by adding TEMPO or NADPH, or SLC7A11 knockdown (Figure 5N; Figure S6E, Supporting Information). SELENOT overexpression alleviated the reduced F‐actin levels, which were prevented by rotenone or BAY‐876 treatment. Overall, our data indicate that SELENOT deficiency leads to NADPH deficiency, which is caused by increases in mtROS levels. This process creates an SLC7A11^high^ state, resulting in abnormal disulfide bonds forming in actin cytoskeleton proteins, followed by reductions in F‐actin levels and detachment of F‐actin from the cytoplasmic membrane. SELENOT overexpression maintains intracellular NADPH production and the redox balance, preventing disulfide ptosis.

We immunoprecipitated endogenous actin proteins in SELENOT‐knockdown cells to further validate the hypothesis that SELENOT deficiency induces disulfide bond formation between actin and other cytoskeletal proteins. The results of Coomassie blue staining showed that SELENOT knockdown induced the coimmunoprecipitation of actin with high‐molecular‐weight proteins near the stacking gel under nonreducing conditions (red box). The coimmunoprecipitation of these proteins shifted under reducing conditions (Figure S7A, Supporting Information). We then analyzed the mass spectrometry of the immunoprecipitated proteins, identifying five pairs of disulfide bonds, including the MYH9 Cys172– Cys511, Cys118– Cys172, Cys739– Cys1379, FLNB Cys51– Cys197, and FLNA Cys428– Cys462 bonds (Figure S7B, Supporting Information).

### 2.6 SELENOT Alleviated Disulfidptosis‐Mediated Skeletal Muscle Atrophy

**Figure 6:**
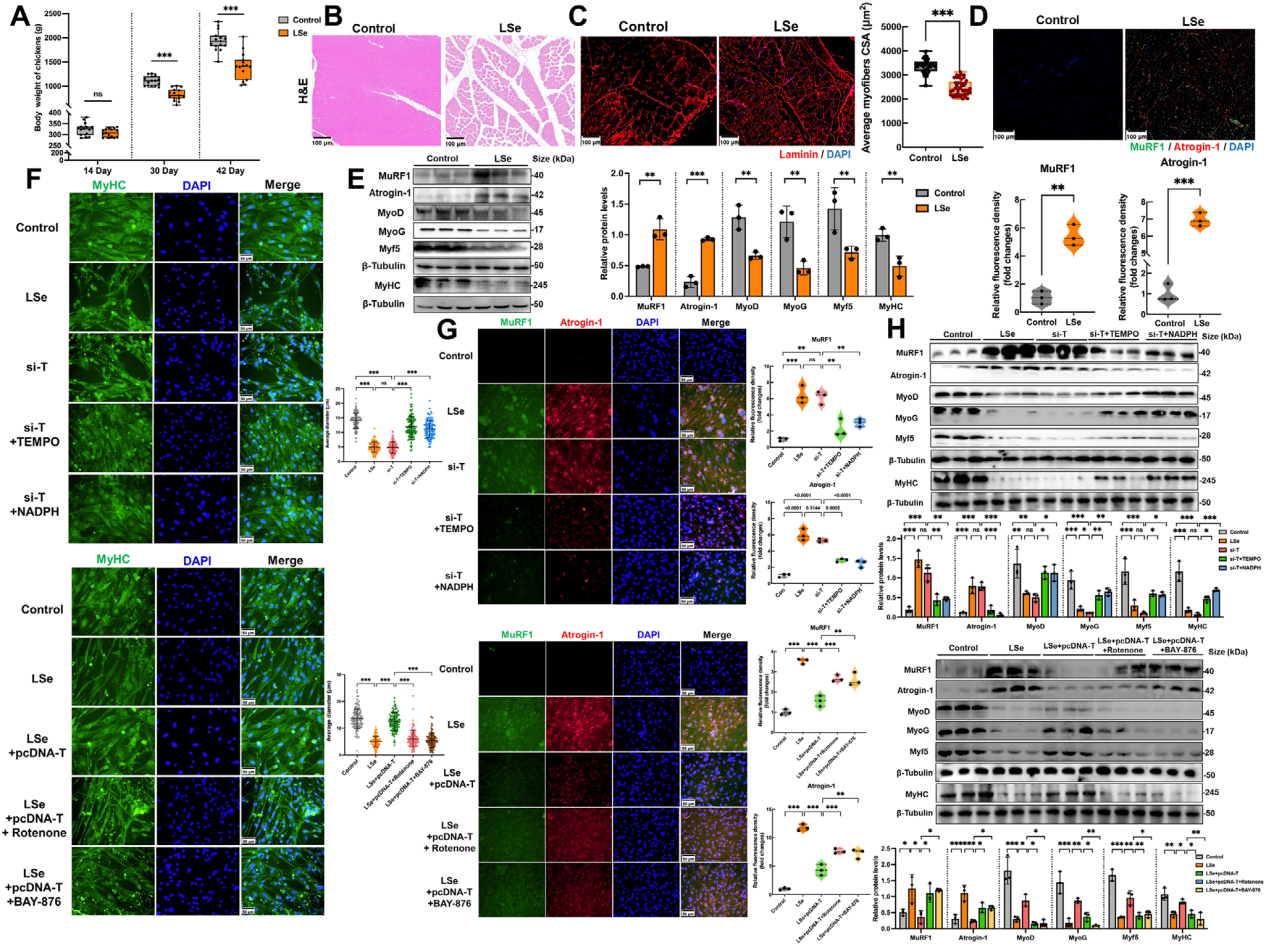
SELENOT reduces skeletal muscle atrophy by inhibiting disulfidptosis. A) Weight change of chickens. *p*‐values were measured by a *t*‐test. *n* = 15. B) Skeletal muscle H&E staining. Scale bar: 100 µm. C) Immunofluorescence staining of Laminin in skeletal muscle tissue and statistical analysis of muscle fiber cross‐sectional area. Scale bar: 100 µm. *p*‐values were measured by a *t*‐test. *n* = 100. D) Skeletal muscle MuRF1 and Atrogin‐1 immunofluorescence staining. Scale bar: 100 µm. *p*‐values were measured by a *t*‐test. *n* = 3. E) Western Blot analysis of skeletal muscle atrophy and differentiation‐related indicators. *p*‐values were measured by a *t*‐test. *n* = 3. F) Changes in myotube diameter of skeletal muscle cells in vitro. Scale bar: 50 µm. *p*‐values were measured by One‐Way ANOVA. *n* = 100. G) MuRF1 and Atrogin‐1 immunofluorescence staining of skeletal muscle cells in vitro. Scale bar: 50 µm. *p*‐values were measured by One‐Way ANOVA. *n* = 3. H) Western Blot analysis of indicators related to skeletal muscle cell atrophy and differentiation in vitro. *p*‐values were measured by One‐Way ANOVA. *n* = 3. The data are shown as the mean ± SDs. ^*^ *p* < 0.05, ^**^ *p* < 0.01, and ^***^ *p* < 0.001.

We evaluated the impact of SELENOT deficiency on chicken body weight during a 42‐day feeding cycle owing to the potential role of abnormal actin cytoskeleton stability in skeletal muscle atrophy. The initial body weights of the chickens were comparable between the groups during the first 14 days (control: 0.32 ± 0.03 kg vs LSe: 0.31 ± 0.02 kg). However, growth was inhibited thereafter in the Se‐deficient chickens, which were 26.8% and 24.6% lighter than control chickens at 30 and 42 days, respectively (**Figure** **6**A; Table S1, Supporting Information). Histopathological examination using hematoxylin and eosin (H&E) staining demonstrated characteristic morphological and structural alterations in the skeletal muscle of Se‐deficient chickens, including myofiber atrophy and disorganization (Figure 6B). Laminin immunofluorescence staining was used to assess the cross‐sectional area of the fibers for precisely quantifying these alterations, which were 28.1% smaller in Se‐deficient chickens (2446.4 ± 315.7 µm^2^) than in the controls (3402.6 ± 334.2 µm^2^). The effect size of morphological alteration was large (Cohen's d = 2.94) with a statistical power of > 0.99 (*n* = 45 per group), confirming the robustness of our findings (Figure 6C; Table S2, Supporting Information). The immunofluorescence and Western blotting results demonstrated that Se deficiency promoted skeletal muscle atrophy through coordinating the activation of catabolic pathways and suppressing regenerative capacity. We observed the marked upregulation of the expression levels of the atrophy markers MuRF1 and Atrogin‐1 in Se‐deficient muscles, which was accompanied by downregulation of the levels of myogenic regulatory factors myoblast determination protein (MyoD), myogenin (MyoG), and myogenic factor 5 (Myf5), and of the structural protein myosin heavy chain (MyHC) (Figure 6D,E).

**Figure 7:**
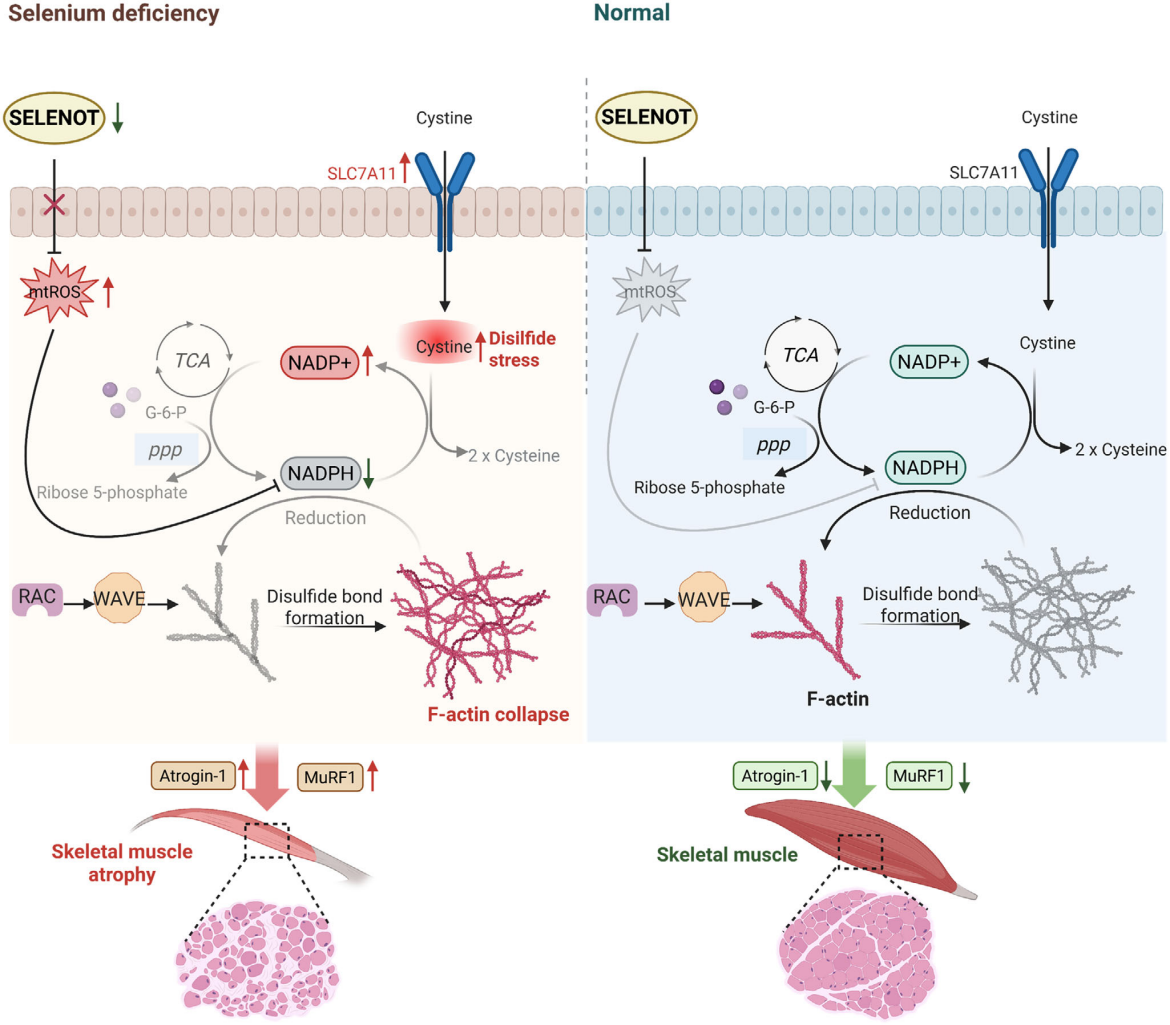
Se deficiency induces actin cytoskeleton collapse and disulfidptosis through the SELENOT/mtROS/NADPH axis, ultimately leading to skeletal muscle atrophy. Image created with BioRender.com, with permission.

We examined whether Se deficiency or SELENOT knockdown affected muscle atrophy in vitro. The muscle fiber diameter in the Se‐deficient and SELENOT knockdown groups was significantly smaller than that in the controls. However, adding TEMPO or NADPH effectively, or SLC7A11 knockdown effectively rescued muscle atrophy in vitro (Figure S8A, Supporting Information). SELENOT overexpression alleviated the muscle cell atrophy caused by Se deficiency, whereas adding rotenone or BAY‐876 eliminated this effect (Figure 6F). The expression levels of muscle atrophy markers (MuRF1 and Atrogin‐1) were markedly higher after Se deficiency or SELENOT knockdown, whereas the protein expression levels of myogenic regulatory factors (MyoD, MyoG, Myf5, and MyHC) were notably lower. These changes were reversed by adding TEMPO or NADPH or SLC7A11 knockdown (Figure S8B,C, Supporting Information). In addition, SELENOT overexpression inhibited the expression levels of MuRF1 and Atrogin‐1 and increased the protein levels of myogenic regulatory factors (MyoD, MyoG, Myf5, and MyHC), reversing the myotube atrophy caused by Se deficiency. However, this process was blocked by adding rotenone or BAY‐876 (Figure 6G,H). These findings demonstrate that Se deficiency induces skeletal muscle atrophy through coordinating the activation of protein degradation pathways and impairing differentiated state maintenance, which are mediated by SELENOT‐deficiency‐triggered disulfidptosis via the mtROS/NADPH axis. SELENOT overexpression exerts protective effects via preventing disulfidptosis and maintaining cytoskeletal protein stability (**Figure** **7**).

## 3 Discussion

Se deficiency causes various clinical and pathological symptoms and is as a key factor in the induction of skeletal muscle disease.^[^ ^26^ ^]^ Se deficiency induces skeletal muscle atrophy through promoting ROS production, resulting in muscle pain and fatigue in patients.^[^ ^27^ ^]^ The biological functions of Se are mainly mediated by selenoproteins.^[^ ^28^ ^]^ We revealed that among the expression of various selenoproteins, SELENOT expression was the most strongly regulated in the skeletal muscle of Se‐deficient chickens. We obtained histological and molecular evidence that SELENOT deficiency induced disulfide accumulation in chicken skeletal muscles through overproducing mtROS, resulting in cytoskeletal protein abnormalities and accelerated muscle mass loss. Furthermore, SELENOT overexpression maintained redox homeostasis, supported NADPH synthesis and metabolism, and effectively prevented Se‐deficiency‐induced muscle atrophy. These findings provide insights into the mechanistic links between SELENOT and skeletal muscle disorders.

The expression levels of all selenoproteins are dependent on Se; however, a hierarchical relationship appears to exist between the sensitivity of different selenoproteins to Se levels. SELENOT more strongly responds to Se deficiency than other proteins in this family.^[^ ^29^ ^]^ Our results support this hypothesis. SELENOT transcription and protein expression levels most strongly regulated the changes in Se content compared with those of the many other selenoproteins. The SELENOT transcription and protein expression levels positively correlated with the Se level in the diet. This indicated that dietary Se deficiency aggravated the loss of SELENOT in the skeletal muscles of chickens. SELENOT is an important membrane‐bound selenoprotein that is mainly located in the mitochondria and endoplasmic reticulum.^[^ ^30^ ^]^ SELENOT has the central structural characteristics of a thioredoxin, leading to strong antioxidant activity.^[^ ^31^ ^]^ We used confocal laser microscopy to confirm that SELENOT predominantly localized to the outer mitochondrial membrane in the skeletal muscle cells of chickens. The results of in vivo and in vitro experiments demonstrated that Se‐deficiency‐induced SELENOT downregulation impaired mitochondrial respiratory chain complex expression, resulting in mtROS overproduction. We assessed the resulting antioxidant enzyme levels, finding that SELENOT deficiency caused the excessive depletion of CAT, SOD1, and SOD2, exacerbating cellular oxidative stress. SELENOT overexpression restored the redox balance through enhancing mitochondrial respiratory chain complex expression. Pharmacological modulation using TEMPO and rotenone confirmed that mtROS directly disrupted mitochondrial respiratory chain complex expression. These findings demonstrate that Se‐deficiency‐induced mitochondrial dysfunction initiates a self‐amplifying cycle in which mtROS overproduction further disrupts redox homeostasis. This finding supports those of previous studies that this redox imbalance exacerbates mitochondrial damage, completing a pathogenic feedback loop.^[^ ^32^ ^]^ The temporal profiling results revealed a well‐defined mechanistic cascade wherein ROS generation in the mitochondria acts as the primary initiator of metabolic dysregulation. The observed sequential pattern of mtROS elevation manifesting prior to NADPH depletion strongly supports oxidative stress as the factor initiating this pathological process rather than a secondary consequence. This causal relationship is further reinforced by the differences in the outcomes in our intervention studies: early antioxidant treatment effectively prevented NADPH depletion; late NADPH supplementation failed to reverse the established oxidative stress.

Glucose serves as the primary substrate for cellular NADPH metabolism and supports NADPH generation through multiple metabolic pathways.^[^ ^33^ ^]^ G6PD catalyzes NADP+ reduction to NADPH via the PPP in the cytoplasm, contributing ≈40% of the cellular‐reducing equivalents.^[^ ^34^ ^]^ The mitochondrial TCA cycle does not directly synthesize NADPH; however, the metabolic intermediates provide the precursors essential for generating NADPH via the citrate–pyruvate shuttle.^[^ ^35^ ^]^ Our in vivo and in vitro experiments revealed that Se deficiency disrupted NADPH synthesis and metabolism in chicken skeletal muscle, which was characterized by lower expression levels of key enzymes in the PPP and citrate–pyruvate shuttle pathways and elevated NADP+/NADPH ratios compared with those in the control. These effects were ameliorated by SELENOT overexpression. Our GO and KEGG analyses demonstrated Se‐ deficiency‐induced downregulation of glucose metabolic pathways, including the PPP, TCA cycle, and carbohydrate response pathway, as ROS function as signaling molecules that regulate metabolic reprogramming.^[^ ^36^ ^]^ The results of our in vitro experiments confirmed that glucose uptake was reduced following SELENOT knockdown, suggesting metabolic reprogramming in the skeletal muscle cells.

We propose that mtROS serve as key mediators of metabolic reprogramming during SELENOT deficiency. The results of pharmacological modulation using mtROS inhibitors and activators confirmed that SELENOT‐deficiency‐induced mtROS overproduction mediates metabolic reprogramming, suppressing the PPP, TCA cycle, and citrate–pyruvate shuttle, ultimately impairing NADPH synthesis. Se deficiency and hypoglycemia are associated in healthy adults.^[^ ^37^ ^]^ Consistent with the finding, we found that serum glucose levels were markedly lower in Se‐deficient chickens, suggesting that the metabolic and oxidative stress effects observed in skeletal muscles owing to Se deficiency contribute to systemic changes in glucose homeostasis. These findings highlight the potential for monitoring muscle‐related effects using serum biomarkers under Se‐deficient conditions.

SLC7A11^high^ cells undergo cell death under low‐glucose conditions through cytoskeletal collapse, which is primarily driven via cytoplasmic cystine accumulation and excessive actin disulfide bond formation following NADPH depletion.^[^ ^10b^ ^]^ Disulfidptosis is driven by a redox imbalance, similar to ferroptosis; yet, the pathways differ in metabolic requirements and depend on iron and lipid peroxidation. Disulfidptosis stems from glucose‐deprivation‐induced NADPH depletion, impairing the reduction in the levels of protein disulfides, especially in cells with high SLC7A11 expression.^[^ ^38^ ^]^ Our findings build on these prior findings by showing that elevated SLC7A11 expression levels in skeletal muscle cells under SELENOT‐deficient conditions. These SLC7A11^high^ muscle cells exhibited enhanced cystine uptake capacity, consistent with prior findings.^[^ ^39^ ^]^ We correspondingly observed increased cysteine and decreased glutamate levels in SELENOT‐deficient chicken skeletal muscle cells in vitro and in vivo compared with controls. SELENOT deficiency also reduced the GSH levels and increased the GSSG levels in the skeletal muscle cells compared with those in the controls. Elevated SLC7A11 expression likely represents an adaptive antioxidant response to mtROS overproduction, which facilitates cystine uptake for synthesizing GSH. However, impaired NADPH metabolism prevents this mechanism from effectively counteracting oxidative stress.

SLC7A11 knockdown in SELENOT‐deficient skeletal muscle cells markedly attenuated disulfidptosis, as indicated by the reduced cytoskeletal collapse and actin disulfide bond formation compared with those in the controls. Specifically, SLC7A11 knockdown alleviated the cystine accumulation and GSH/GSSG reduction as well as inhibited disulfide bond formation between the RAC/WAVE complex and the actin cytoskeleton compared with those in the controls, reversing the F‐actin detachment from the plasma membrane. The pharmacological modulation of mtROS and NADPH further supported the role of the SELENOT/mtROS/NADPH axis in regulating the cystine metabolism in Se‐deficient skeletal muscles. The RAC/WAVE complex, a crucial mediator of disulfidptosis, requires all components for actin disulfide bond assembly.^[^ ^40^ ^]^

The reasons for the occurrence of disulfidptosis in skeletal muscle remain unclear; however, our study provides additional evidence. We used in vitro and in vivo models to find that SELENOT deficiency promoted RAC/WAVE complex formation, leading to abnormal actin disulfide bonding, cytoskeletal contraction, and membrane detachment. These findings establish a role for SELENOT in skeletal muscle disulfidptosis. We confirmed that the SELENOT/mtROS/NADPH axis mediates skeletal muscle disulfidation through targeted modulation using TEMPO, rotenone, BAY‐876, and NADPH. Se deficiency can induce various forms of programmed cell death, such as apoptosis, necroptosis, and autophagy^.[^ ^41^ ^]^ These diverse cell death pathways are primarily attributed to disrupted redox homeostasis and increased oxidative stress caused by Se insufficiency. Additionally, Se deficiency downregulates GPX4 expression, inducing ferroptosis through the accumulation of lipid peroxidation products.^[^ ^42^ ^]^ However, we found that Se deficiency triggers disulfidopathy in skeletal muscle cells, in contrast to the aforementioned mechanisms. This finding not only broadens the understanding of the diversity of cell death mechanisms induced by Se deficiency but also highlights the unique role of the SELENOT/mtROS/NADPH axis in regulating disulfidptosis in skeletal muscle cells.

The proliferation, differentiation, and fusion of skeletal muscles are regulated through myogenic regulatory factors, such as MyHC, Myf5, MyoD, and MyoG.^[^ ^43^ ^]^ Atrogin‐1 and MuRF1 are two E3 ubiquitin ligases involved in protein degradation and are molecular markers of muscle atrophy.^[^ ^44^ ^]^ SELENOT deficiency impairs muscle cell proliferation and promotes apoptosis.^[^ ^41a^ ^]^ We observed the upregulation of Atrogin‐1 and MuRF1 levels, along with the downregulation of MyHC, Myf5, MyoD, and MyoG levels in the SELENOT‐deficient skeletal muscle cells of chickens compared with those in the controls. Additionally, SELENOT deficiency reduced the chicken body weight, although this effect was not notable before 14 days of age. These findings indicated that SELENOT deficiency exacerbated skeletal muscle atrophy and inhibited proliferation and differentiation in chickens. These adverse effects may be associated with abnormal cytoskeletal disulfide bond formation. We propose that SELENOT‐deficiency‐induced disulfidptosis is a key mechanism underlying skeletal muscle atrophy in chickens given the importance of actin cytoskeleton stability in skeletal muscle function.^[^ ^45^ ^]^ Treating the si‐T group with TEMPO or NADPH reversed the SELENOT‐knockdown‐induced muscle atrophy, whereas administering rotenone or BAY‐876 reversed the protective effects of SELENOT overexpression in the LSe+pcDNA‐T group. Furthermore, SLC7A11 knockdown suppressed MuRF1 and Atrogin‐1 expression levels; elevated MyHC, MyoD, MyoG, and Myf5 protein levels; and attenuated myotube atrophy in SELENOT‐deficient cells, reinforcing the link between SLC7A11‐mediated disulfidptosis and muscle atrophy. These data show that the SELENOT/mtROS/NADPH axis mediates disulfide‐induced skeletal muscle atrophy. The regulatory role of SELENOT in skeletal muscle atrophy warrants further investigation to determine the underlying mechanisms.

Although our data were primarily derived from chicken models and primary avian cells, cross‐species protein sequence conservation analysis (Figure S9, Supporting Information) supports the evolutionary conservation of these mechanisms, suggesting their relevance to mammals and humans. The chicken model is suitable for Se deficiency studies, featuring thoroughly pathologies such as exudative diathesis and white muscle disease, which mimic aspects of human nutritional deficiencies such as Keshan disease.^[^ ^46^ ^]^ The chicken model offers advantages over zebrafish, including rapid growth, low cost, and skeletal muscle composition akin to humans. Zebrafish are more suitable in embryonic imaging and genetic manipulation but lack the muscle mass and behavioral complexity of avian or mammalian systems.^[^ ^47^ ^]^ This study advances our understanding of the pathogenesis of skeletal muscle atrophy in chickens. The results require validation in mammalian or human systems in future studies.

We determined the role of SELENOT in Se‐deficiency‐induced skeletal muscle atrophy in chickens. SELENOT was the most strongly regulated selenoprotein in response to dietary Se levels. SELENOT levels inversely correlated with mtROS and NADPH levels, demonstrating protective effects through maintaining actin cytoskeletal stability and preventing muscle atrophy. These findings provide insight into the molecular mechanisms linking Se deficiency to skeletal muscle atrophy in chickens. This study advances the understanding of skeletal muscle atrophy pathogenesis and provides perspectives for developing therapeutic strategies against muscle wasting disorders.

## 4 Experimental Section

### Animals and Experimental Design

All animal procedures were performed in compliance with the National Research Council Guidelines for the Care and Use of Laboratory Animals and were approved by the Animal Care and Use Committee of the Northeast Agricultural University (SRM‐11). A total of 60 one‐day‐old chickens from the Xinghua Breeding Farm (Harbin, China) were randomly allocated into two groups (*n* = 30 per group), each subdivided into three parallel subgroups. The control group was fed a diet containing 0.278 mg kg^−1^ Se, whereas the Se‐deficient group was fed a diet containing 0.008 mg kg^−1^ Se. The basal diet consisted of corn and soybean meal from Se‐deficient areas in Longjiang County, Heilongjiang Province, and was supplemented with Na_2_SeO_3_ as the control group. The detailed feed compositions and nutritional levels are listed in Table S3 (Supporting Information). The body weight of the chickens was recorded weekly. Chickens had ad libitum access to feed and water (without Se supplementation) under controlled environmental conditions: temperature was maintained at 25 ± 1.5 °C, relative humidity was 60–70%, oxygen concentration was >19.5%, carbon dioxide levels were <0.5%, and ammonia levels were <10 ppm. The chickens were randomly selected for sampling and euthanasia at 42 days old. Leg muscles were collected at 4 °C, divided into appropriate‐sized tissue blocks, and either fixed in 4% paraformaldehyde for histopathological analysis or snap‐frozen in liquid nitrogen and stored at –80 °C until the subsequent experiments.

### Isolation of Primary Myoblasts

The primary myoblasts were isolated according to established protocols. Briefly, 12‐day‐old chicken embryos were sterilized with alcohol, followed by extracting the embryos and dissecting the skeletal muscle. Additional methodological details are provided in the Supporting Information.

### Transfection

SELENOT (gene ID: 425041) was overexpressed by constructing the pcDNA3.1(+)‐FLAG‐SELENOT (pcDNA‐T) plasmid via PCR amplification, sequence verification, and subsequent cloning into the pcDNA3.1(+) vector. SELENOT and SLC7A11 were knocked down using commercially available siRNA‐SELENOT (si‐T) and si‐SLC7A11 plasmids from RiboBio (Guangzhou, China). All plasmids were transfected using Lipofectamine 2000 (Thermo Fisher Scientific). The primer sequences and transfection complex preparations are provided in Tables S4 and S5 (Supporting Information), respectively.

### Cell Treatment with Se and Reagents

The myotube differentiation medium containing horse serum was replaced with the basal medium supplemented with Se following differentiation to establish the treatment groups. Cells were cultured for 5 days under distinct Se conditions: the control group was maintained in medium containing 5 ng mL^−1^ sodium selenite to maintain physiological Se levels, the LSe group was cultured in sodium Se‐free medium (0 ng mL^−1^) to establish chronic Se deprivation,^[^ ^48^ ^]^ and the control group received medium containing 5 ng mL^−1^ sodium selenite to mimic physiological Se levels (medium composition detailed in Table S6, Supporting Information). Experiments were performed after 24 h of treatment, TEMPO (HY‐112879), mitochondrial ROS activator (HY‐B1756), NADPH inhibitor BAY‐876 (HY‐100017), and NADPH (HY‐113324) were obtained from MedChemExpress and were used according to the manufacturer's protocols (Table S7, Supporting Information).

### Histology and IHC

Muscle samples were fixed in 4% paraformaldehyde for 24 h, processed via graded ethanol dehydration, embedded in paraffin, sectioned, and stained with H&E. Cell morphology was analyzed using a microscope (Thermo Fisher Scientific). Muscle fiber number and diameter were quantified using ImageJ software by tracing the fiber boundaries. A minimum of 50 intact muscle fibers per sample were analyzed, excluding fibers with an incomplete sarcolemma. All quantifiable fibers in the cross‐sectional images were included in the analysis. Details on IHC analyses are provided in the Supporting Information. Information on the primary and secondary antibodies used for the IHC analysis is provided in Table S8 (Supporting Information).

### IF

Tissue sections were subjected to xylene dewaxing, followed by ethanol gradient rehydration, for IF analysis. The protocols are detailed in the Supporting Information, and specific information regarding the IF antibodies is presented in Table S8 (Supporting Information).

### Periodic Acid‐Schiff (PAS) Stain

Tissue sections were stained using PAS reagent. The frozen slices were removed from a −20 °C refrigerator and allowed to equilibrate to room temperature before processing. The Supporting Information provides further details.

### Glucose Uptake Assay

Glucose uptake was assessed via incubating cells with 300 µm 2‐(N‐(7‐nitrobenz‐2‐oxa‐1,3‐diazol‐4‐yl)amino)‐2‐deoxyglucose (2‐NBDG; Thermo Fisher Scientific, Carlsbad, CA, USA) in glucose‐free phosphate‐buffered saline (PBS) for 45 min. The cells were washed three times following incubation with glucose‐free PBS to remove the unincorporated dye. The cells were then imaged using a fluorescence microscope (Nikon, Japan). Fluorescence intensity was measured at 480 nm excitation and 550 nm emission wavelengths.

### Fluorescent Staining of ROS

ROS levels were assessed using dihydroethidium (DHE) staining (S0063, Beyotime, China). Tissue sections were incubated with DHE solution at 37 °C in the dark for 30 min following the manufacturer's protocol. We detected cellular ROS levels via treating cells with serum‐free medium and incubated the cells with either 10 µm DCFH‐DA or 10 µm DHE for 30 min. Fluorescence was imaged using a Nikon microscope (Nikon, Tokyo, Japan).

### MitoSOX Staining

The cells were washed three times with Hank's Balanced Salt Solution (Solarbio, China) according to the manufacturer's instructions. The Supporting Information provides additional details.

### Flow cytometry

Cells were washed three times with Hank's Balanced Salt Solution (Solarbio, China) and then incubated with 1 µm MitoSOX Red (Invitrogen, USA) at 37 °C for 20 min. Mitochondrial ROS levels were analyzed using a FACSCelesta flow cytometer (BD Biosciences, New Jersey, USA).

### Mitochondrial Respiratory Complex Activity

Mitochondria were isolated from fresh skeletal muscle tissues or cultured cells through differential centrifugation at 700 ×*g* for 10 min to remove debris and nuclei, followed by centrifugation at 10, 000 × g for 15 min to pellet the mitochondria, in ice‐cold mitochondrial isolation buffer (250 mm sucrose, 10 mm HEPES, and 1 mm EGTA, pH 7.4) supplemented with protease inhibitors. The protein concentrations of the mitochondrial preparations were determined using the Bradford assay (Beyotime, Shanghai, China). The MRCC enzymatic activities were measured using standardized commercial assay kits (Abbkine, Wuhan, China), with absorbance readings acquired using a multimode microplate reader (Thermo Fisher Scientific) in accordance with the manufacturer's specifications.

### OCR and ECAR Measurements

Metabolics were profiled using an XFp extracellular flux analyzer (Agilent Technologies) to quantify mitochondrial respiration and glycolytic activity in the primary skeletal muscle cells. Cells were plated at 2 × 10⁴ cells/well in specialized XF96 microplates. Glycolytic capacity was assessed by measuring the ECAR following sequential challenge with glucose (10 mm), the ATP synthase inhibitor oligomycin (1 µm), and the glycolytic blocker 2‐DG (50 mm). Mitochondrial function was evaluated by monitoring the OCR after sequential exposure to oligomycin (1 µm), the uncoupler FCCP (2 µm), and electron transport chain inhibitors rotenone and antimycin A (0.5 µm each). All metabolic parameters were derived using proprietary analysis software.

### Analysis of Amino Acid Composition

The amino acid composition was evaluated by collecting three muscle or myoblast samples from each treatment group. Each sample was homogenized in 1 mL of physiological saline and sonicated for 3 min, followed by centrifugation at 16740 ×g for 20 min at 4 °C. The supernatant was then filtered through a 0.22 µm membrane. The filtered samples were analyzed using an amino acid analyzer (Hitachi LA8080).

### NADP^+^ and NADPH Measurement

The NADPH and total NADP (NADPH + NADP^+^) levels in the muscle tissues and cells were quantified according to the manufacturer's instructions. Briefly, 0.1 g of muscle tissue or cells from a 6‐well plate were lysed in 300 µL extraction buffer (20 mm nicotinamide, 20 mm NaHCO_3_, and 100 mm Na_2_CO_3_) for 10–30 min. Details are provided in the Supporting Information.

### Detection of GSH/GSSG System

Muscle tissue (0.1 g) or cells from a 6‐well plate were homogenized in 0.9% NaCl. The GSH and GSSG levels were measured according to the instructions of the manufacturer (Nanjing Jiancheng Bioengineering Research Institute, A061‐2‐1). The samples were gently agitated to ensure reagent mixing, and then the absorbance was measured at 405 nm using a microplate reader (Thermo Fisher Scientific).

### Fluorescent Staining of Actin Filaments and Cellular Membrane

The treated cells were washed three times with PBS and then fixed overnight with 4% paraformaldehyde at 4 °C. The Supporting Information provide additional details.

### Transcriptome Sequencing

The total RNA was isolated from chicken skeletal muscle using TRIzol reagent. RNA concentration and purity were assessed using a NanoDrop spectrophotometer (Thermo Fisher Scientific) and a LabChip GX Touch HT nucleic acid analyzer (PerkinElmer, Waltham, MA, USA). The high‐quality RNA samples were processed by Wuhan Biotechnology Co., Ltd. (Wuhan, China) for preparing and sequencing the cDNA library. mRNA was enriched using oligo(dT) beads. RNA sequencing libraries were constructed according to the manufacturer's protocol for an Illumina KAPA RNA Seq kit. Sequencing was performed using the Illumina NovaSeq platform. Differential gene expression was analyzed using DESeq2, with significance thresholds set at |log_2_(fold change) | > 1 and *P* < 0.05.

### Proteomic Analysis

Proteomics was conducted according to established protocols based on quantitative mass spectrometry. Details are provided in the Supporting Information.

### Immunoprecipitation and Disulfide Bond Identification

Cell lysates were subjected to affinity purification with an antiactin antibody overnight. Subsequently, the eluted products were divided into two aliquots, one of which was supplemented with β‐mercaptoethanol at a final concentration of 2% for the reducing treatment. The gels were separated via SDS‐PAGE under reducing or nonreducing conditions and stained with Coomassie Blue. The bands were excised and subjected to in‐gel tryptic digestion. Refer to the Supplementary Materials for detailed procedures regarding disulfide bond identification.

### Western Blot Analysis

Separated proteins were transferred onto nitrocellulose membranes in Tris buffer containing 20% methanol. Membranes were blocked with 5% skim milk at 37 °C for 2.5 h, followed by overnight incubation with primary antibodies at 4 °C. The membranes were washed with TBST and then were incubated with a horseradish‐peroxidase‐conjugated goat antirabbit IgG secondary antibody (1:10000, ABclonal, China) for 70 min. Protein bands were visualized using an ECL detection kit (Biosharp, China) and imaged using an exposure analyzer (TransGen Biotech, Beijing, China). The band intensity was quantified using ImageJ software. Detailed information on the primary antibodies is provided in Table S8 (Supporting Information).

### Statistical Analysis

All the data were obtained from at least three independent biological replicates. Statistical analyses were performed using GraphPad Prism 9.0 (GraphPad Software Inc., San Diego, CA, USA). Data pre‐processing included evaluation for outliers using the ROUT method (Q = 1%) in GraphPad Prism 9.0. Unpaired two‐tailed Student's *t*‐tests were used for two‐group comparisons. Multiple‐group comparisons were analyzed using one‐way analysis of variance (ANOVA), followed by Tukey's or Dunnett's post hoc tests. Complex experimental designs were evaluated using two‐way ANOVA with Tukey's multiple comparison test. The normality of the data distribution was confirmed using the D'Agostino–Pearson test in GraphPad Prism 9.0. Statistical significance was set at *p* < 0.05. Images were analyzed using Image‐Pro Plus 6.0 and GraphPad Prism 9.0 software.

### Compliance with Ethics Requirements

All procedures used in this research were approved by the Institutional Animal Care and Use Committee of Northeast Agricultural University (SRM‐11).

## Conflict of Interest

The authors have announced no conflict of interest. All authors have read the manuscript and consented to submit it in its current form for consideration for publication in the Journal.

## Author's Contribution

H.L. contributed to conceptualization, data curation, and writing of the original draft. H.W. contributed to conceptualization and methodology. Z.Z. was responsible for investigation and validation. S.X. contributed to data curation and formal analysis. C.Z. oversaw project administration. T.X. provided resources, administered the project, acquired funding, and contributed to writing – review and editing.

## Supporting information



Supporting Information

## Data Availability

The data that support the findings of this study are available from the corresponding author upon reasonable request.
