# Supplementary material for: Redox Cascade in Chicken Skeletal Muscle: SELENOT Suppression in Selenium Deficiency Triggers Disulfidptosis via mtROS‐NADPH Dysregulation
Source: Adv Sci (Weinh). 2025 Sep 15;12(45):e07283. doi: 10.1002/advs.202507283 (PMC12677588; doi:10.1002/advs.202507283)
Supplement: Supplementary file 1 — Supporting Information [file ADVS-12-e07283-s001.docx]

***Supporting Information***

**Redox Cascade in Chicken Skeletal Muscle: SELENOT Suppression in Selenium Deficiency Triggers Disulfidptosis via mtROS-NADPH Dysregulation**

*Huanyi Liu ^a,b,c^, Hao Wu ^a,b,c^, Ziyu Zhang ^a,b,c^, Shiwen Xu ^a,b,c^, Cong Zhou ^a,b^, Tong Xu ^a,b*^*

^a^ College of Veterinary Medicine, Northeast Agricultural University, Harbin 150030, P.R. China.

^b^ Key Laboratory of the Provincial Education Department of Heilongjiang for Common Animal Disease Prevention and Treatment, College of Veterinary Medicine, Northeast Agricultural University, Harbin, 150030, P.R. China.

^c^ Laboratory of Embryo Biotechnology, College of Life Science, Northeast Agricultural University, Harbin, 150030, P.R. China.

****Corresponding author:***

Address: Northeast Agricultural University, Harbin, 150030, P. R. China (Tong Xu).

E-mail address: tongxu@neau.edu.cn

**Contents of this file**

The Supporting Information includes the following information:

**Experimental Section (Page S2-S5)**

**Supplementary Tables (Table S1-8, Page S6-S10)**

**Supplementary Figure (Figure S1-S9, Page S11-18)**

**Experimental Section**

**Isolation of primary myoblasts**

The tissue was washed 2-3 times with D-HANKS solution, followed by careful removal of fascia and cartilage. The muscle tissue was then minced into small fragments. D-HANKS was added to achieve a final volume of 10 mL, and the mixture was allowed to settle for 3-5 minutes before supernatant removal. Subsequently, 1% type I collagenase (Bioshap) was added for enzymatic digestion at 37℃ for 15 min. The digested tissue was sequentially filtered through 70 μm and 40 μm cell strainers. The resulting filtrate was centrifuged at 1000 rpm for 10 minutes, and the supernatant was discarded. The cell pellet was resuspended in DMEM/F12 medium (Gibco) supplemented with 20% fetal bovine serum (Gibco), thoroughly mixed, and plated in six-well culture plates. The cultures were maintained in a humidified incubator at 37℃ with 5% CO_2_. Cell purification was performed using the differential adhesion method. Upon reaching 80% confluence, the culture medium was replaced with differentiation medium containing 2% horse serum (HBS; Procell) to induce myotube formation.

**Histology and immunohistochemistry (IHC)**

For IHC analysis, tissue sections were subjected to deparaffinization and rehydration using standard protocols, followed by antigen retrieval in EDTA buffer at 62℃ overnight. Endogenous peroxidase activity was quenched by treatment with 3% H_2_O_2_ solution, and non-specific binding sites were blocked using immunohistochemistry rapid blocking buffer for 15 min. The sections were then incubated with primary antibodies at 4℃ overnight, followed by incubation with appropriate secondary antibodies at room temperature for 1 h. Antigen-positive cells were visualized using a DAB substrate kit according to the manufacturer's instructions.

**Immunofluorescence (IF).**

Tissue sections were subjected to overnight antigen retrieval treatment. Subsequently, the sections were permeabilized with 0.2% Triton X-100 for 15 min to enhance membrane permeability, followed by blocking with rapid immunohistochemical blocking buffer for 15 min. For cell samples, fixation was performed using 4% paraformaldehyde, after which the cells were permeabilized with 0.1% Triton X-100 for 10 minutes and blocked with 5% bovine serum albumin (BSA). Both tissue sections and cell samples were incubated with primary antibodies at 4°C overnight. Following three washes with phosphate-buffered saline (PBS), the samples were incubated with fluorescently labeled secondary antibodies in the dark at room temperature for 1 h.

**Periodic Acid-Schiff (PAS) stain**

Tissue sections were fixed with tissue fixative for 15 min and subsequently rinsed under running tap water. Following the protocol provided by the reagent kit (Wuhan Xinwei Biotechnology Co., Ltd.), the sections were sequentially immersed in PAS staining solution, differentiated with hydrochloric acid aqueous solution, and treated with ammonia water for blue color restoration, followed by a final rinse with tap water. After dehydration through an ethanol series, the sections were mounted and examined under a microscope for image acquisition and subsequent analysis. In the stained tissue sections, carbohydrate components were visualized as magenta, while cell nuclei appeared light blue.

**MitoSOX staining**

Cells were incubated with 100 nM MitoTracker Green (Invitrogen, USA) at 37°C for 20 min in the dark. Following one wash with Hank's balanced salt solution (Solarbio, China), 1 μM MitoSOX Red (Invitrogen, USA) was added and incubated at 37°C for 30 min under light-protected conditions. Fluorescence imaging was performed using a confocal microscope (TCS SP8, Leica) with excitation/emission wavelengths of 396/610 nm for MitoSOX and 488/510 nm for MitoTracker, respectively. Quantitative analysis of fluorescence intensity was conducted using ImageJ software (National Institutes of Health, USA).

**NADP^+^ and NADPH measurement**

Following centrifugation of the cell lysate, the supernatant was aliquoted into two 150 μL samples. One aliquot was heated at 60°C for 30 min to selectively degrade NADP^+^ while preserving NADPH, followed by immediate cooling on ice. The second aliquot was maintained at 4°C for total NADP determination. For the assay, 20 μL of each sample (heated or unheated) was transferred to a 96-well plate and combined with 80 μL of NADP cycling buffer containing glucose-6-phosphate dehydrogenase (Sigma Aldrich, USA). After dark incubation at 30°C, 20 μL of 10 mM glucose-6-phosphate was added to each well, and absorbance at 570 nm was measured at 1-min intervals for a minimum of 6 min using a microplate reader. The NADP^+^ concentration was determined by calculating the difference between total NADP and NADPH levels.

**Fluorescent staining of actin filaments and cellular membrane**

Fixed cells were permeabilized with permeabilization buffer containing 1% Triton X-100 for 5 min at room temperature, followed by three washes with phosphate-buffered saline (PBS). The cells were then incubated with F-actin antibody (1:200 dilution, Abcam, UK) at room temperature for 2 h. After three additional PBS washes, the cells were incubated with Dylight 594-conjugated goat anti-rabbit IgG (1:1000 dilution, Biodragon, China) in the dark at room temperature for 30 min. Finally, cells were stained with CellMask Green Plasma Membrane Stain (Thermo Fisher Scientific, USA) for 30 min. Fluorescence imaging was performed using a confocal microscope (TCS SP8, Leica).

**Proteomic sequencing**

Skeletal muscle tissue (0.1 g) was homogenized in lysis buffer (8 M urea, 200 mM HEPES pH 8.5, 1× Roche protease inhibitor, 1× Roche phosphatase inhibitor) using a 21-gauge needle with 20 strokes, yielding a protein concentration of approximately 5 mg/mL. The homogenate was centrifuged at 20,000 × g for 5 min at 4°C. Protein reduction was performed using 5 mM dithiothreitol (DTT) at 37°C for 25 min, followed by alkylation with 10 mM iodoacetamide in the dark at room temperature for 30 minutes. Excess iodoacetamide was quenched with 15 mM DTT in the dark at room temperature for 15 min. Protein digestion was initiated with LysC protease (1:100 enzyme-to-protein ratio) at 37°C for 3 h, followed by trypsin digestion (1:100 enzyme-to-protein ratio) overnight at 37°C. TMT reagents were dissolved in anhydrous acetonitrile and added to peptides at a 1:2 reagent-to-peptide ratio. After 1 h of incubation at room temperature, the reaction was quenched with hydroxylamine. All TMT-labeled samples were combined in a 1:1 ratio and fractionated using a high-pH reverse-phase peptide fractionation kit, yielding 12 fractions with different acetonitrile concentrations. These fractions were pooled into six samples, concentrated by vacuum centrifugation, desalted, and resuspended in 5% acetonitrile with 5% formic acid for LC-MS/MS analysis. Mass spectrometry data were processed using Sequest software, with database searches performed against the Mus musculus UniProt database. Search parameters included a precursor ion tolerance of 50 ppm and a product ion tolerance of 1 Da. TMT tags and carbamidomethylation were set as fixed modifications, while methionine oxidation was considered as a variable modification. Peptide spectrum matches (PSMs) were filtered to a 1% false discovery rate (FDR), and quantification was performed by summing reporter ion intensities. Protein abundance was normalized such that the total signal intensity for each protein across all channels equaled 100, enabling relative abundance measurements.

**Immunoprecipitation and disulfide bond identification**

To identify the protein components within the excised bands, MS/MS raw data were processed with MaxQuant software (version 1.6.6.0), employing default label-free quantification parameters and intensity-based absolute quantification (iBAQ) for precise abundance estimation. For the detection of native disulfide bonds, MS/MS raw data were analyzed using pLink2 software (version 2.4.4), followed by manual validation of disulfide-linked peptide spectra to confirm bond localization45. In a parallel experiment designed to validate disulfide linkages, non-reduced tryptic peptides were divided into two aliquots. One aliquot was reduced with 2 mM DTT at 55 °C for 30 min, alkylated with 6 mM iodoacetamide (IAA) at 25 °C in the dark for 30 min, and quenched with 10 mM cysteine for 30 min; the other aliquot remained untreated. Following desalting, both samples were subjected to the aforementioned mass spectrometry analysis. The resulting raw data files were searched with pLink2 to identify native disulfides, while Mascot (version 2.6.0) was used to characterize peptides liberated from disulfides upon DTT reduction, ensuring robust confirmation of inter- and intra-molecular bonds.

**Table S1.** Body weight Statistical Power Analysis of Control vs LSe groups.

| **Age (days)** | **Control (kg)** | **LSe (kg)** | **Mean difference (kg)** | **Effect**  **size (d)** | **Statistical power** | ***p*-value** |
| --- | --- | --- | --- | --- | --- | --- |
| 14 | 0.32 ± 0.03 | 0.31 ± 0.02 | 0.01 | 0.36 | 0.18 | 0.0713 |
| 30 | 1.12 ± 0.09 | 0.82 ± 0.12 | 0.30 | 2.50 | >0.99 | <0.001 |
| 42 | 1.91 ± 0.25 | 1.44 ± 0.28 | 0.47 | 1.78 | 0.99 | <0.001 |

**Table S2:** Muscle fiber characteristics Statistical Power Analysis of Control vs LSe groups

| **Parameter** | **Control** | **LSe** | **Mean Difference** | | **Effect Size (d)** | **Statistical Power** | ***p-*value** |
| --- | --- | --- | --- | --- | --- | --- | --- |
| CSA (μm²) | 3402.6±334.2 | 2446.4±315.7 | | 956.2 | 2.94 | >0.99 | <0.001 |

**Table S3**. Basal diet composition

| **Component** | **Control group (g)** | **LSe group (g)** |
| --- | --- | --- |
| corn | 80520 | 80520 |
| soybean | 26400 | 26400 |
| bean cake | 19800 | 19800 |
| Premix | 46200 | 46200 |
| vitamin | 39.6 | 39.6 |
| Trace elements (excluding Se) | None | 198 |
| Trace elements (including Se) | 198 | None |
| methionine | 132 | 132 |
| lysine | 396 | 396 |

**Table S4.** The sequences of siRNA

| **Name** | **Sequences** |  |
| --- | --- | --- |
| siRNA-SELENOT | F (5’-3’): GGGUGUUCGAGGAGUACAUTT |  |
|  | R (5’-3’): AUGUACUCCUCGAACACCCTT |  |
| siRNA-SLC7A11 | F (5’-3’): ACUAUAAUUAUCAGAAUUGCU |  |
|  | R (5’-3’): CAAUUCUGAUAAUUAUAGUCC |  |
| siRNA-NC | F (5’-3’): UUCUCCGAACGUGUCACGUTT |  |
|  | R (5’-3’): ACGUGACACGUUCGGAGAATT |  |

**Table S5.** The preparation method for cell transfection complexes

| **Reagent** | **Dose (μL)** |  |
| --- | --- | --- |
| Lipofectamine 2000 | 3 |  |
| Opti serum-free culture medium | 47 |  |
| si-NC/siRNA-SELENOT/siRNA-SLC7A11/ pCDNA3.1/pCDNA3.1- SELENOT | 5 |  |
| Opti serum-free culture medium | 45 |  |
| Total | 100 |  |

**Table S6.** Composition of culture media formulations.

| Component | Control | LSe |
| --- | --- | --- |
| DMEM/F12 (Gibco) | + | + |
| FBS (1%, Gibco) | + | + |
| Transferrin (Sigma, 11,096-37-0) | 5 μg/mL | 5 μg/mL |
| Insulin (Sigma, 12,584-58-6) | 10 μg/mL | 10 μg/mL |
| Sodium selenite (Sigma, S5261) | 5 ng/mL | - |

**Table S7.** Drug treatment conditions

| **Compound** | **Concentration** | **Treatment Duration** | **Catalog no.** |
| --- | --- | --- | --- |
| TEMPO | 10 μM | 12 h | HY-112879 |
| BAY-876 | 50 nM | 24 h | HY-100017 |
| Rotenone | 100 nM | 24 h | HY-B1756 |
| NADPH | 1 mM | 24 h | HY-113324 |

**Table S8.** Antibody information

| **Antibodies** | **Use in this study** | **Concentration** | **Company** | **Catalog no.** |
| --- | --- | --- | --- | --- |
| SELENOT | WB/IHC/IF | 1:500/1:100/1:100 | Bioss | bs-4459R |
| Total OXPHOS | WB | 1:1000 | Abcam | ab110413 |
| catalase | WB | 1:1000 | Abcam | ab209211 |
| SOD1 | WB | 1:1000 | ABclonal | A12537 |
| SOD2 | WB | 1:800 | ABclonal | A21805 |
| 4-HNE | IHC | 1:50 | Abcam | ab48506 |
| Hexokinase II | WB | 1:1000 | ABclonal | A22319 |
| LDHA | WB | 1:500 | Wanleibio | WL03271 |
| G6PD | WB/IF | 1:1000/1:200 | ABclonal | A1537 |
| PGLS | WB | 1:1000 | ABclonal | A8366 |
| PGD | WB | 1:1000 | ABclonal | A0563 |
| IDH1 | WB/IF | 1:1000/1:150 | ABclonal | A24133 |
| IDH2 | WB | 1:500 | Wanleibio | WL01963 |
| MDH1 | WB | 1:1000 | ABclonal | A9673 |
| α-KG | WB | 1:1000 | FineTest | FNab04518 |
| ME2 | WB | 1:1000 | ABclonal | A9650 |
| CS | WB | 1:1000 | ABclonal | A5713 |
| SLC7A11 | WB | 1:1000 | ABclonal | A2413 |
| RAC | WB | 1:1000 | Wanleibio | WL02851 |
| WAVE | WB | 1:1000 | ABclonal | A19601 |
| NCKAP1 | WB | 1:1000 | ABclonal | A12229 |
| CYFIP1 | WB | 1:500 | ABclonal | A20924 |
| ABI2 | WB | 1:1000 | ABclonal | A14992 |
| MYH9 | WB | 1:1000 | ABclonal | A0173 |
| TLN1 | WB | 1:1000 | ABclonal | A4158 |
| FLNA | WB | 1:3000 | ABclonal | A3738 |
| FLNB | WB | 1:1000 | Absin | abs116088 |
| Drebrin | WB | 1:1000 | ABclonal | A6366 |
| F-actin | WB/IF | 1:500/1:25 | Bioss | bs-1571R |
| Laminin | IF | 1:100 | ABclonal | A27860 |
| MuRF1 | WB/IF | 1:1000/1:200 | ABclonal | A3101 |
| Atrogin-1 | WB/IF | 1:1000/1:200 | ABclonal | A3699 |
| MyoD | WB | 1:1000 | Wanleibio | WL04662 |
| MyoG | WB | 1:1000 | Wanleibio | WL01132 |
| Myf5 | WB | 1:1000 | ABclonal | A16227 |
| MyHC | WB/IF | 1:1000/1:50 | ABclonal | A25357 |
| β-Tubulin | WB | 1:10000 | Abclonal | A12289 |

**Figure S1.**


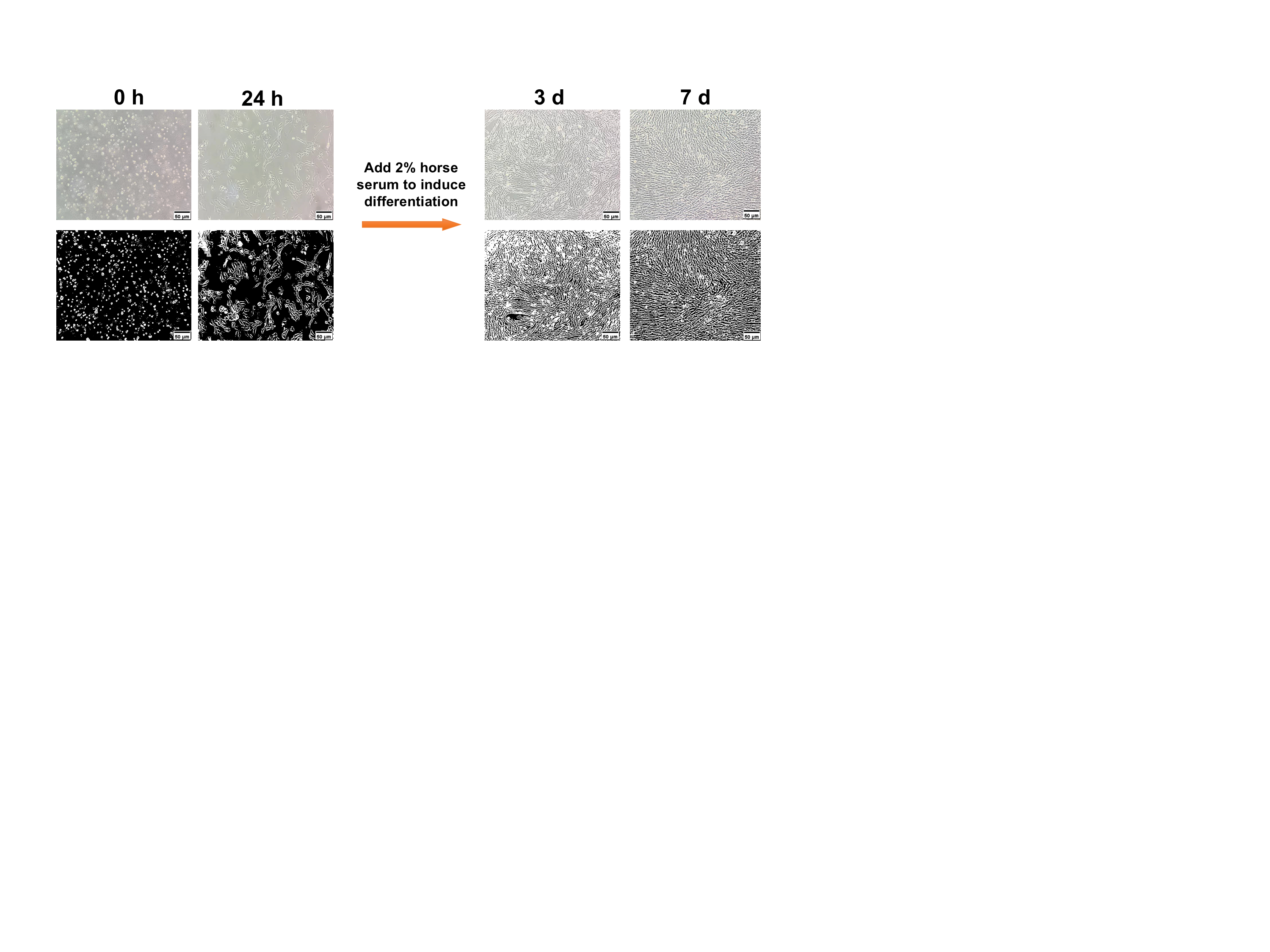


Figure S1. Morphological changes after extraction and differentiation of primary skeletal muscle cells from chicken embryos. Scale bar: 50 μm.

**Figure S2.**


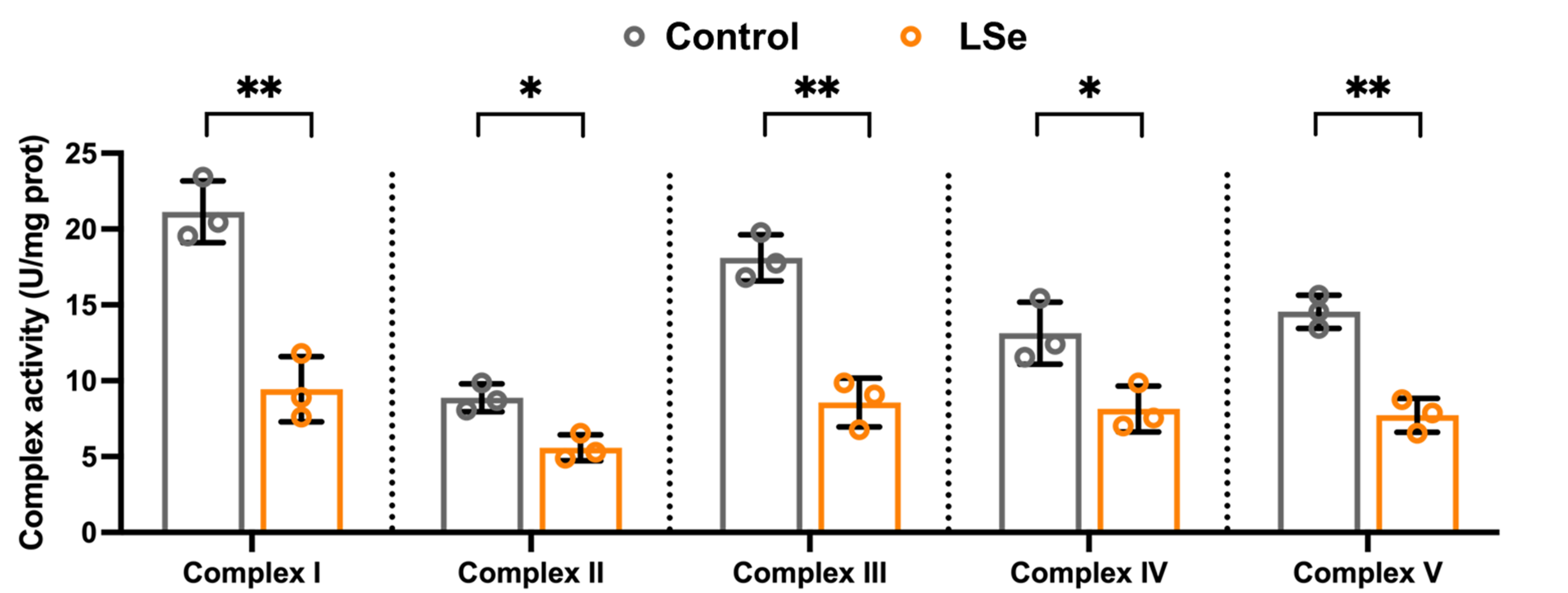


Figure S2. The changes of mitochondrial Complex I-Complex IV activity in skeletal muscle. *p*-values were measured by t-test. n=3. The data are shown as the mean ± SDs. **p* < 0.05, ***p* < 0.01, and ****p* < 0.001.

**Figure S3.**


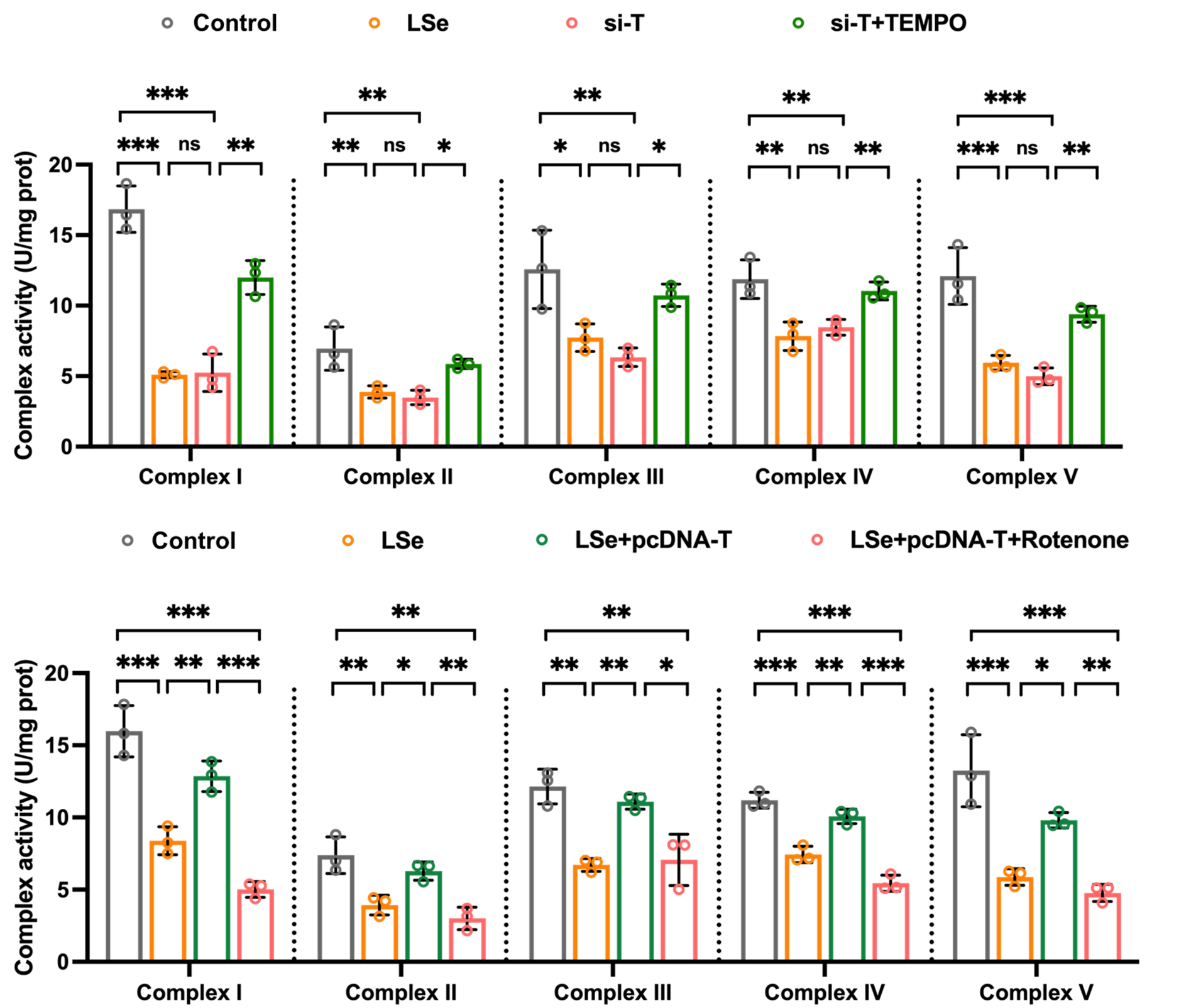


Figure S3. The changes of mitochondrial Complex I-Complex IV activity in skeletal muscle cells. *p*-values were measured by One-Way ANOVA. n=3. The data are shown as the mean ± SDs. **p* < 0.05, ***p* < 0.01, and ****p* < 0.001.

**Figure S4.**


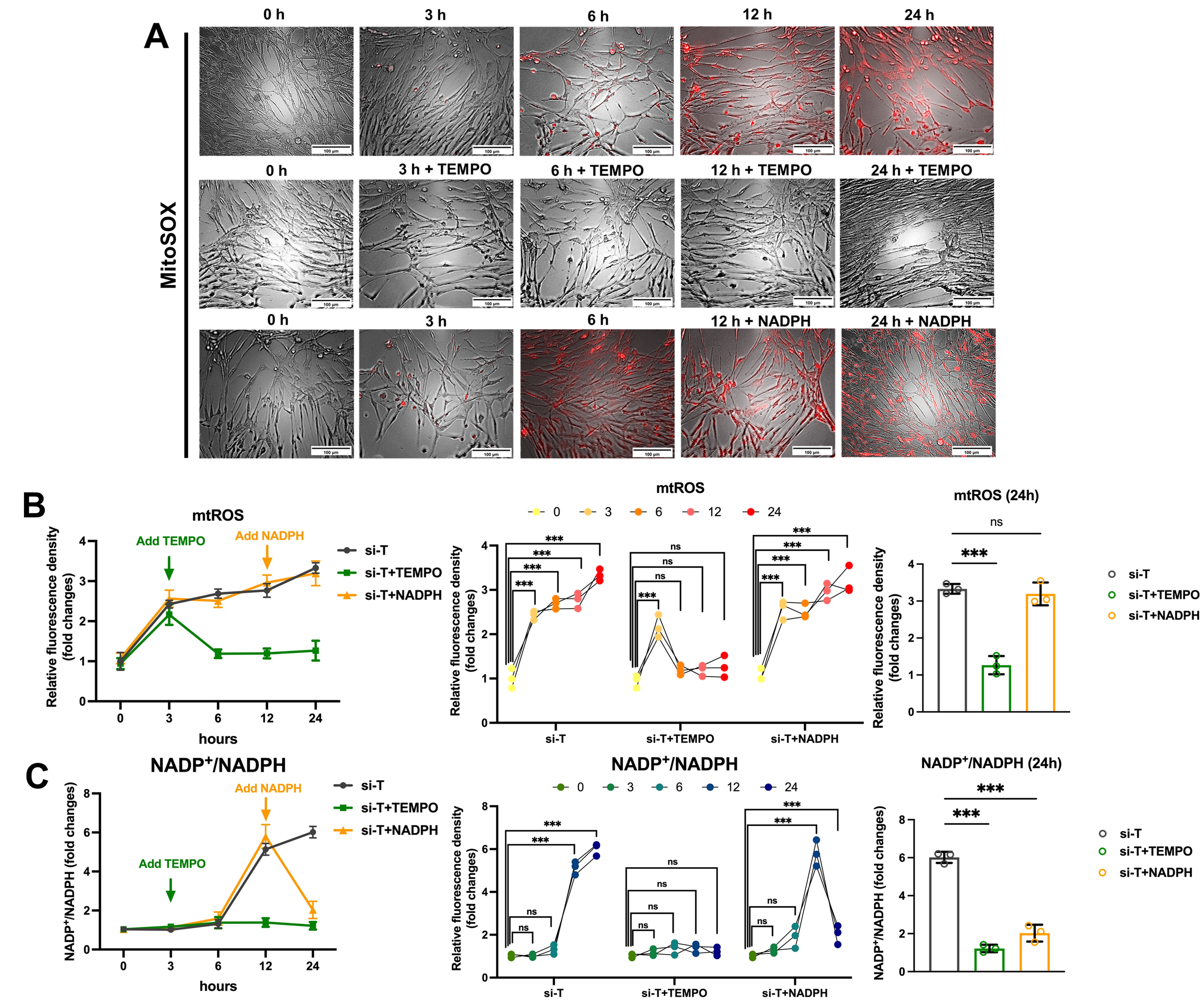


Figure S4. Temporal profiling reveals a definitive redox cascade initiated by SELENOT deficiency. (A) mtROS fluorescence staining of skeletal muscle cells in vitro. Scale bar: 100 μm. (B) Quantitative analysis of intracellular mtROS fluorescence intensity over time following SELENOT knockdown, early-phase (3h) TEMPO treatment, and late-phase (12h) NADPH supplementation. *p*-values were measured by One-Way ANOVA. n=3. (C) Temporal changes in NADP+/NADPH ratio after SELENOT knockdown, early-phase (3h) TEMPO treatment, and late-phase (12h) NADPH supplementation. *p*-values were measured by One-Way ANOVA. n=3. The data are shown as the mean ± SDs. **p* < 0.05, ***p* < 0.01, and ****p* < 0.001.

**Figure S5.**


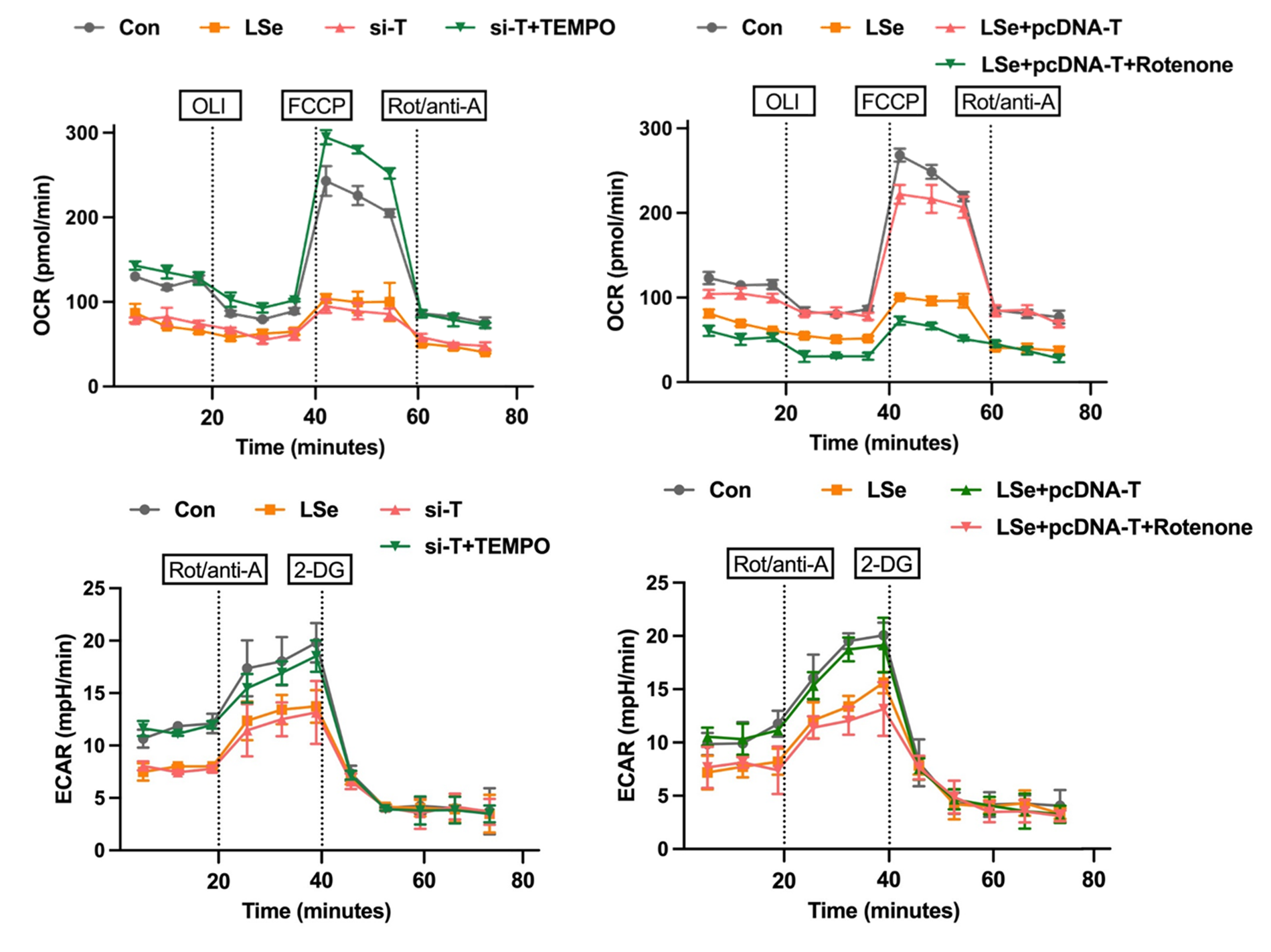


Figure S5. Metabolic profiling of skeletal muscle cells across treatment groups was performed by assessing oxygen consumption rate (OCR) and extracellular acidification rate (ECAR) using Seahorse XF technology. n=3.

**Figure S6.**


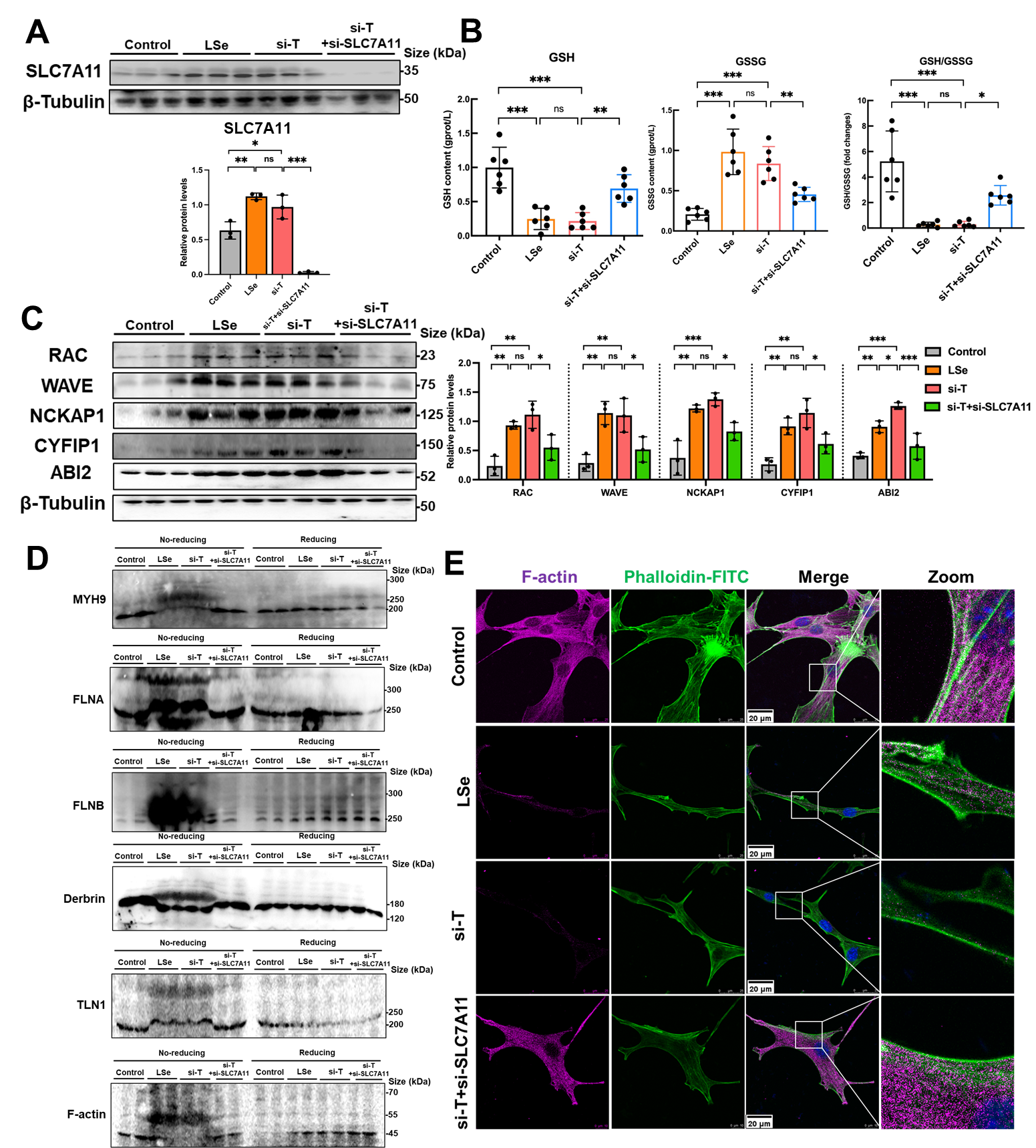


Figure S6. SLC7A11 knockdown ameliorates disulfidptosis induced by SELENOT deficiency. (A) Western Blot analysis of SLC7A11 in skeletal muscle cells in vitro. *p*-values were measured by One-Way ANOVA. n=3. (B) The content of GSH/GSSG in skeletal muscle cells in vitro. *p*-values were measured by One-Way ANOVA. n=6. (C) Western Blot analysis of RAC/WAVE pathway related indicators in vitro skeletal muscle cells. *p*-values were measured by One-Way ANOVA. n=3. (D) Formation of disulfide bonds in the cytoskeleton of skeletal muscle cells, n=4. (E) F-actin staining of skeletal muscle cells in vitro. Scale bar: 20 μm. The data are shown as the mean ± SDs. **p* < 0.05, ***p* < 0.01, and ****p* < 0.001.

**Figure S7.**

**
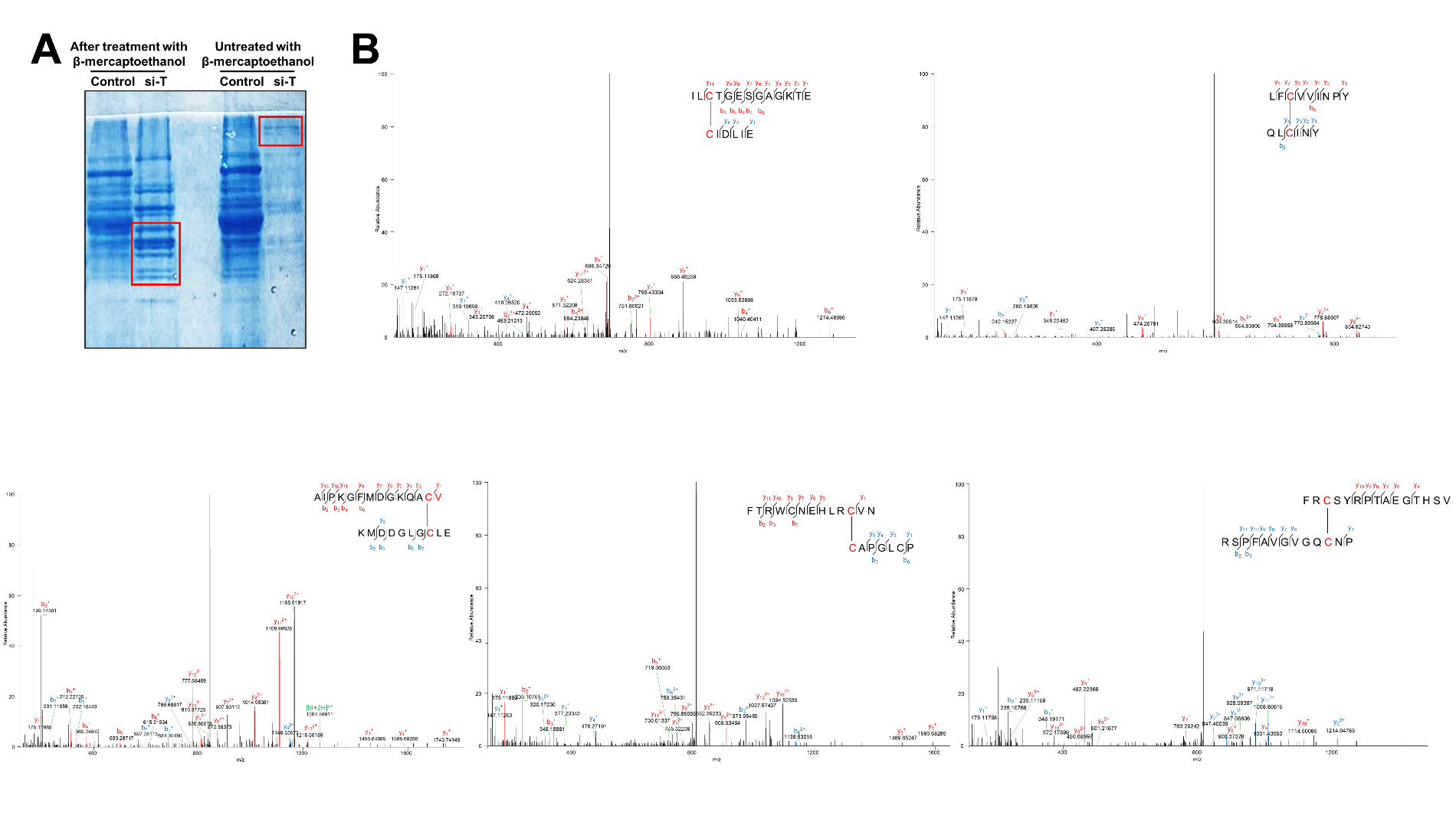
**

Figure S7. Immunoprecipitation of proteins interacting with actin. (A) Coomassie blue-stained. (B) The annotated MS/MS spectrum. All experiments were independently repeated at least three times with similar results.

**Figure S8.**


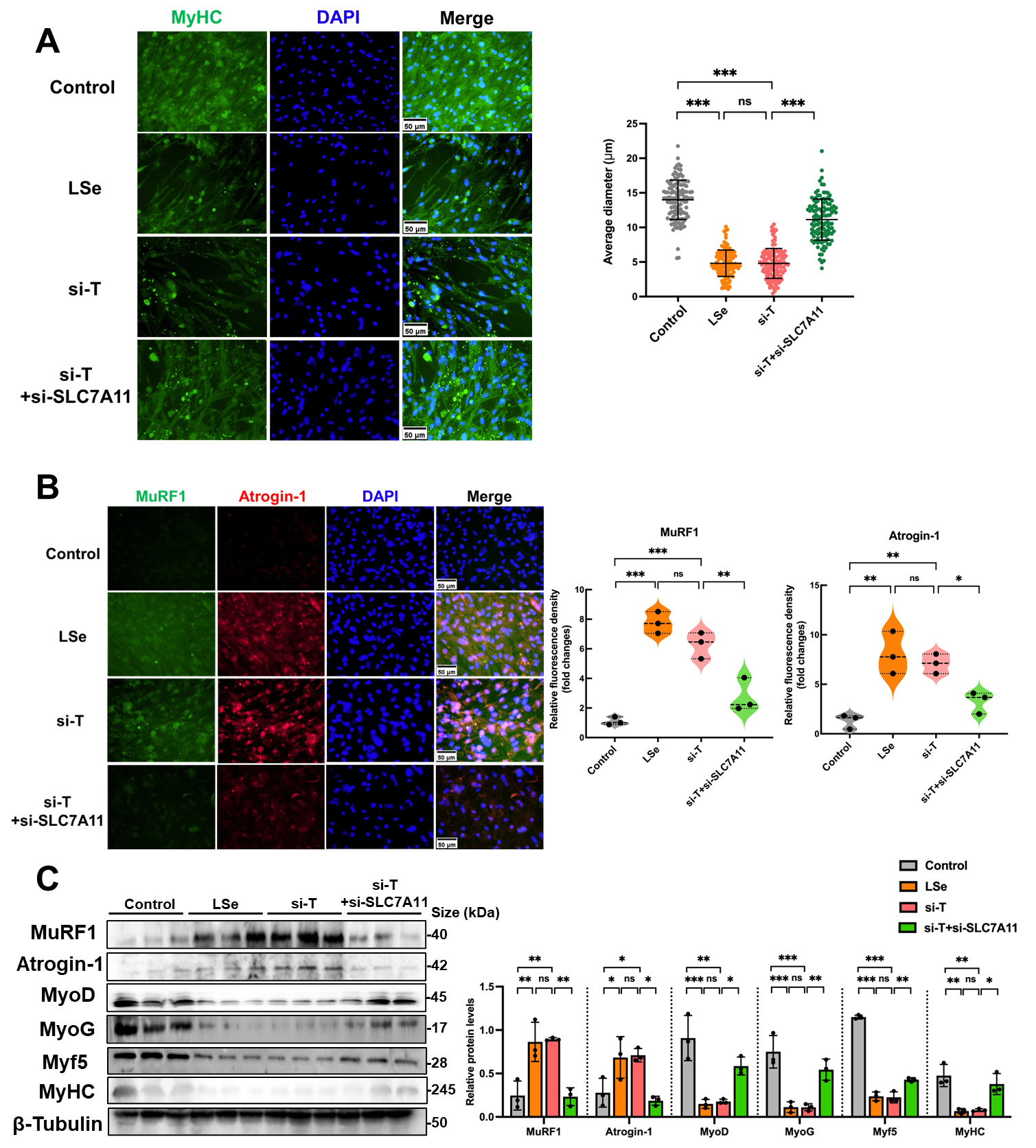


Figure S8. SLC7A11 knockdown can alleviate skeletal muscle cell atrophy induced by SELENOT deficiency. (A) Changes in myotube diameter of skeletal muscle cells in vitro. p-values were measured by One-Way ANOVA. n=100. (B) MuRF1 and Atrogin-1 immunofluorescence staining of skeletal muscle cells in vitro. Scale bar: 100 μm. *p*-values were measured by One-Way ANOVA. n=3. (C) Western Blot analysis of indicators related to skeletal muscle cell atrophy and differentiation in vitro. *p*-values were measured by One-Way ANOVA. n=3. The data are shown as the mean ± SDs. **p* < 0.05, ***p* < 0.01, and ****p* < 0.001.

**Figure S9.**


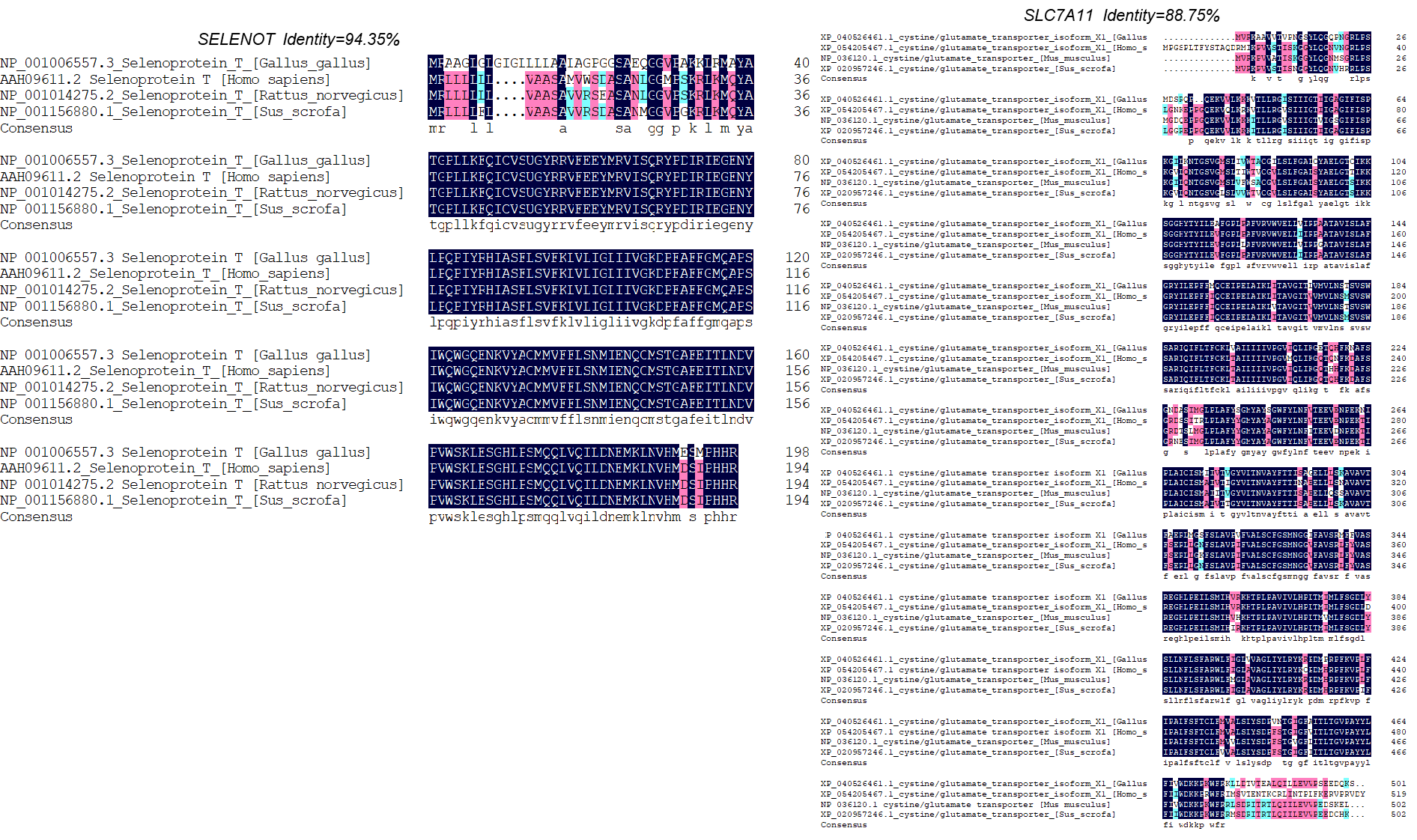


Figure S9. Conservation of Amino Acid Sequences in SELENOT and SLC7A11 Proteins.
